# Enhancing transcription–replication conflict targets ecDNA-positive cancers

**DOI:** 10.1038/s41586-024-07802-5

**Published:** 2024-11-06

**Authors:** Jun Tang, Natasha E. Weiser, Guiping Wang, Sudhir Chowdhry, Ellis J. Curtis, Yanding Zhao, Ivy Tsz-Lo Wong, Georgi K. Marinov, Rui Li, Philip Hanoian, Edison Tse, Salvador Garcia Mojica, Ryan Hansen, Joshua Plum, Auzon Steffy, Snezana Milutinovic, S. Todd Meyer, Jens Luebeck, Yanbo Wang, Shu Zhang, Nicolas Altemose, Christina Curtis, William J. Greenleaf, Vineet Bafna, Stephen J. Benkovic, Anthony B. Pinkerton, Shailaja Kasibhatla, Christian A. Hassig, Paul S. Mischel, Howard Y. Chang

**Affiliations:** 1 Department of Pathology, Stanford University School of Medicine, Stanford, CA USA; 2 Sarafan ChEM-H, Stanford University, Stanford, CA USA; 3 Center for Personal Dynamic Regulomes, Stanford University, Stanford, CA USA; 4 Department of Genetics, Stanford University School of Medicine, Stanford, CA USA; 5 Boundless Bio, San Diego, CA USA; 6 Medical Scientist Training Program, University of California, San Diego, La Jolla, CA USA; 7 Department of Dermatology, Stanford University School of Medicine, Stanford, CA USA; 8 Department of Chemistry, Pennsylvania State University, University Park, PA USA; 9 Department of Computer Science and Engineering, University of California, San Diego, La Jolla, CA USA; 10 Department of Medicine, Stanford University School of Medicine, Stanford, CA USA; 11 Stanford Cancer Institute, Stanford University School of Medicine, Stanford, CA USA; 12 Howard Hughes Medical Institute, Stanford University School of Medicine, Stanford, CA USA

**Keywords:** Cancer genetics, Cancer therapy, Cancer

## Abstract

Extrachromosomal DNA (ecDNA) presents a major challenge for cancer patients. ecDNA renders tumours treatment resistant by facilitating massive oncogene transcription and rapid genome evolution, contributing to poor patient survival^1–7^. At present, there are no ecDNA-specific treatments. Here we show that enhancing transcription–replication conflict enables targeted elimination of ecDNA-containing cancers. Stepwise analyses of ecDNA transcription reveal pervasive RNA transcription and associated single-stranded DNA, leading to excessive transcription–replication conflicts and replication stress compared with chromosomal loci. Nucleotide incorporation on ecDNA is markedly slower, and replication stress is significantly higher in ecDNA-containing tumours regardless of cancer type or oncogene cargo. pRPA2-S33, a mediator of DNA damage repair that binds single-stranded DNA, shows elevated localization on ecDNA in a transcription-dependent manner, along with increased DNA double strand breaks, and activation of the S-phase checkpoint kinase, CHK1. Genetic or pharmacological CHK1 inhibition causes extensive and preferential tumour cell death in ecDNA-containing tumours. We advance a highly selective, potent and bioavailable oral CHK1 inhibitor, BBI-2779, that preferentially kills ecDNA-containing tumour cells. In a gastric cancer model containing *FGFR2* amplified on ecDNA, BBI-2779 suppresses tumour growth and prevents ecDNA-mediated acquired resistance to the pan-FGFR inhibitor infigratinib, resulting in potent and sustained tumour regression in mice. Transcription–replication conflict emerges as a target for ecDNA-directed therapy, exploiting a synthetic lethality of excess to treat cancer.

## Abstract (web summary)

Extrachromosomal DNA makes cancerous tumours resistant to treatment, but this research demonstrates that increasing transcription–replication conflict allows for targeted elimination of cancer cells containing extrachromosomal DNA, and thus sustained tumour regression in mice.

## Main

Extrachromosomal DNAs (ecDNAs) are a frequent mechanism for oncogene amplification in diverse cancer types and are associated with worse patient outcomes than other kinds of focal amplification^1,2^. ecDNAs can arise during the transition to, development and progression of cancers, and they exhibit unique biological features that provide fitness advantages to malignant cells^3^. The acentric structure of ecDNA facilitates random segregation, highly elevated copy number, intratumoural genetic heterogeneity and rapid tumour evolution^1,4,5^, contributing to aggressive tumour growth and therapeutic resistance^6,7^. The circular topology of ecDNAs also profoundly alters transcription^8,9^. ecDNAs exhibit highly accessible chromatin and increased oncogene expression compared to non-circular amplifications, even after controlling for DNA copy number^1,10–12^. Further, ecDNAs can cluster in the nucleus to generate new, functional enhancer–promoter interactions both in *cis* and in *trans*^10,12^. Earlier studies showed that ecDNAs highly transcribe annotated protein-coding genes^11^, but it is unclear whether the full landscape of RNA transcription—such as intergenic, antisense or other long non-coding RNAs—is altered. ecDNA exhibits open chromatin and is marked by active histone modifications such as H3K27ac and H3K4me3 (refs. ^1,11,13,14^), raising the possibility of a more permissive transcriptional environment. We hypothesized that the highly accessible chromatin of ecDNA could generate a therapeutically exploitable vulnerability.

## Rampant transcription on ecDNA

**Fig. 1:**
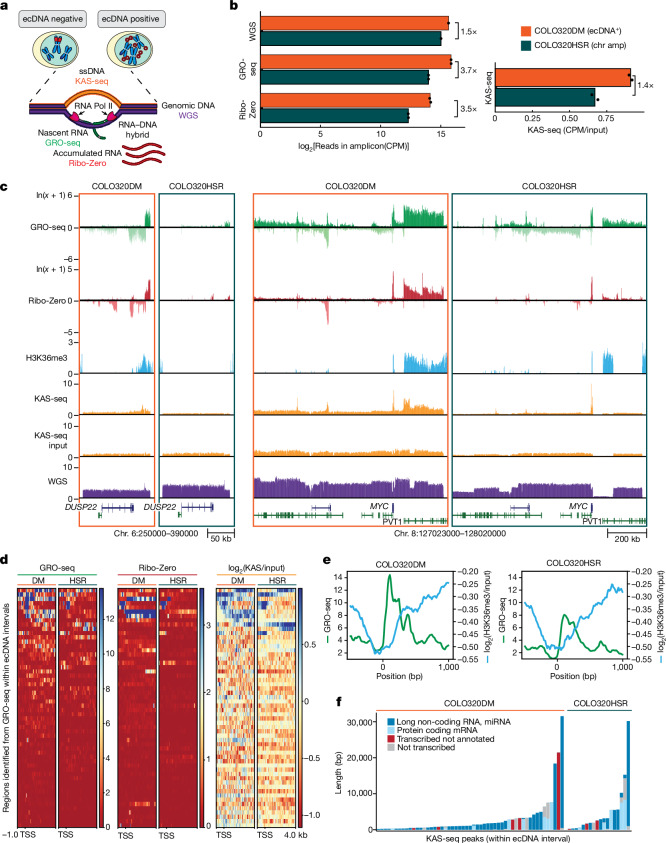
Pervasive transcription on ecDNA drives ssDNA accumulation. **a**, Schematic of relevant genomic assays. **b**, Read density of genomic assays in COLO320DM and COLO320HSR in total counts per million (CPM) within the ecDNA intervals (amplicon boundaries defined in Extended Data Fig. 1). KAS-seq read density is shown as CPM of the KAS-seq relative to CPM of the input of total DNA after fragmentation but before biotin enrichment for ssDNA signals. The mean of two biological replicates is shown for GRO-seq, Ribo-Zero and KAS-seq; a single replicate is shown for WGS. **c**, Genome tracks highlighting two regions within the ecDNA interval. H3K36me3 chromatin immunoprecipitation followed by sequencing (ChIP–seq) is displayed as log_2_ of input-normalized coverage. **d**, Metagene heatmap plot visualization of GRO-seq, Ribo-Zero RNA sequencing (RNA-seq) and log_2_ of input-normalized coverage of KAS-seq within the ecDNA interval. All plots are anchored at the transcription start site (TSS) of combined transcribed regions as identified by HOMER using both biological replicates of GRO-seq in COLO320DM and COLO320HSR. **e**, Metagene plot showing GRO-seq and H3K36me3 ChIP–seq coverage within the ecDNA interval. All plots are anchored at the GRO-seq TSS as identified by HOMER using both biological replicates. H3K36me3 ChIP–seq coverage is displayed as log_2_(H3K36me3/input). **f**, KAS-seq peaks from two biological replicates in the ecDNA interval annotated by transcription status according to GRO-seq data and annotation status according to Gencode v.43. One representative biological replicate for each condition is visualized for **c**, **d** and **e**. chr amp, chromosomal amplification; RNA Pol II, RNA polymerase II.

**Extended Data Fig. 1:**
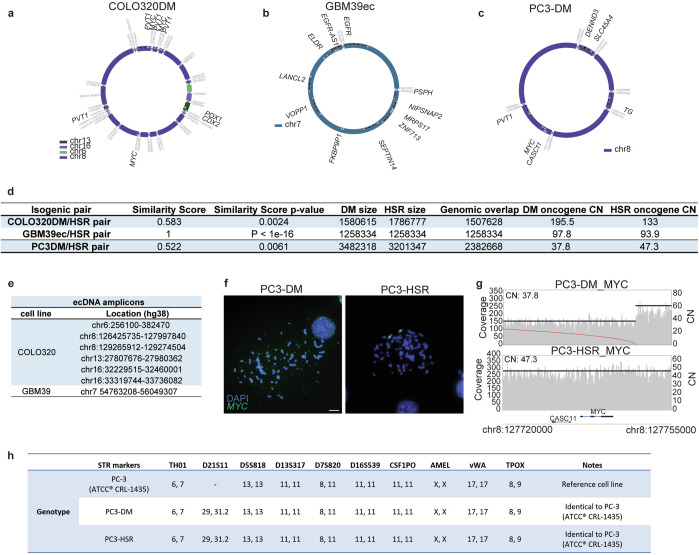
Characterization of isogenic cell line pairs. (**a**-**c**) The structure of one dominant ecDNA detected in COLO320DM (a), GBM39ec (b) and PC3-DM (c). COLO320DM ecDNA reconstruction is adapted from Hung, et al. Nature 2021^10^ using hg38 coordinates; GBM39ec ecDNA reconstruction is adapted from Turner, et al. Nature 2017^2^ using hg38 coordinates; PC3-DM ecDNA was reconstructed by short-read WGS aligned to hg38, capturing the MYC-containing genome cycle identified by AmpliconArchitect. Some complexities of the PC3 DM ecDNA may not be captured from short read sequencing alone. (**d**) Amplicon similarity analysis of 3 near-isogenic cell line pairs as computed from whole genome sequencing data. Major oncogene copy number was extracted for individual cell line: COLO320 pairs: *MYC*; GBM39 pairs: *EGFR*; PC3: *MYC*. (**e**) Genomic intervals in COLO320 and GBM39 cells used in this study for ecDNA amplicons. (**f**) Representative metaphase-FISH images of PC3-DM and PC3-HSR cells confirming *MYC* gene amplification on ecDNA and chromosome respectively. For each clone, about 6–15 metaphase spread images were collected from a one-off validation experiment to check amplicon status. Scale bar: 10 µm (**g**) Amplicon structure and absolute gene copy of *MYC* between PC3-DM and PC3-HSR based on WGS. (**h**) Comparison of STR profiles between PC-3 from ATCC, PC3-DM and PC3-HSR.

To test this hypothesis, we performed global run-on sequencing (GRO-seq)^15^ and ribosmoal RNA (rRNA)-depleted RNA sequencing (Ribo-Zero) to profile nascent transcription and accumulated RNAs, respectively (Fig. 1a), providing a comprehensive landscape of RNA biogenesis from ecDNAs. To control for the effects of focal amplification and assess ecDNA-specific transcriptional changes, we focused on a pair of isogenic colorectal cancer cell lines derived from the same patient: COLO320DM (*MYC* amplification on ecDNA, also called double minute (DM)) and COLO320HSR (chromosomal *MYC* amplification on homogeneously staining region (HSR)), which are nearly matched for amplicon copy number as revealed by whole-genome sequencing (WGS) (Extended Data Fig. 1a,d)^11^. Notably, COLO320DM showed a nearly 4-fold increase in nascent RNA and accumulated RNA read density from ecDNA, beyond the level expected from differences in amplicon copy number compared to COLO320HSR (Fig. 1b).

**Extended Data Fig. 2:**
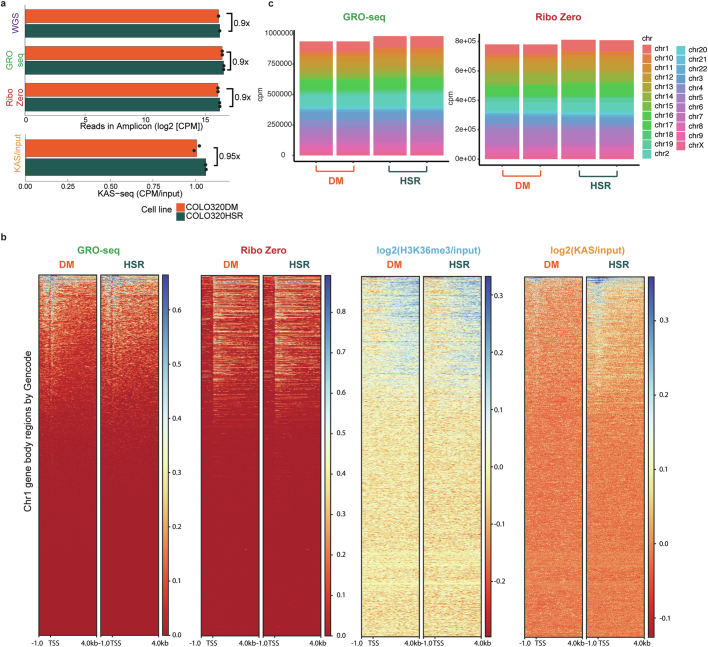
Similar levels of transcription and ssDNA accumulation on normal chromosome 1 of COLO320 cell lines. (**a**) Read density of genomic assays in COLO320DM and COLO320HSR in total counts per million (CPM) on chromosome 1, which is outside of ecDNA intervals. KAS-seq read density is shown as (CPM) of the KAS-seq relative to CPM of the input. The mean of two biological replicates is shown for GRO-seq, Ribo-Zero and KAS-seq; a single replicate is shown for WGS. (**b**) Metagene heatmap plot visualization of GRO-seq, Ribo Zero RNA-seq, log2 of input-normalized H3K36me3 ChIP-seq, and log2 of input-normalized coverage of KAS-seq within chromosome 1. All plots are anchored at the GRO-seq TSS as identified by HOMER. One representative biological replicate for each condition is visualized. (**c**) Stacked bar charts of read density in total counts per million (CPM) of GRO-seq and Ribo-Zero in COLO320DM and COLO320HSR across all chromosomes.

**Extended Data Fig. 3:**
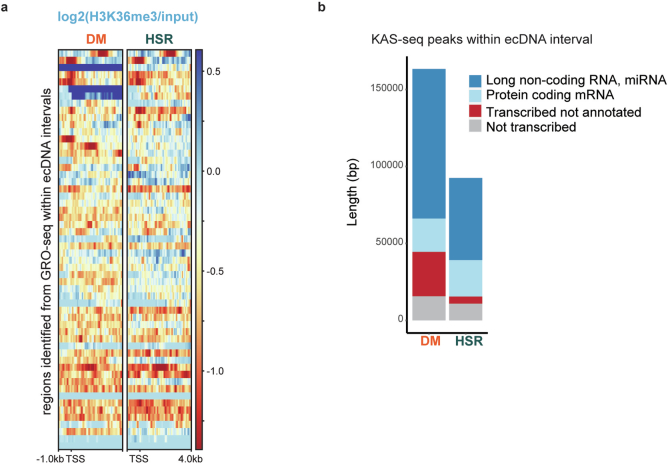
H3K36me3 and KAS-seq signals within the ecDNA interval of COLO320 cell lines. (**a**) Metagene heatmap plot visualization of log2 of input-normalized H3K36me3 ChIP-seq within the ecDNA interval. Plots are anchored at the GRO-seq TSS as identified by HOMER. One representative biological replicate for each cell line is visualized. (**b**) Accumulative bar plots of length distributions of all KAS-seq peaks identified within the ecDNA interval classified by GRO-seq transcription status and Gencode v43 annotation. Two biological replicates were used per cell line.

The increase in transcription was not limited to the *MYC* oncogene but was pervasive across the entire ecDNA, including non-coding, antisense and numerous previously unannotated transcripts (Fig. 1c,d). This widespread increase in transcription is specific to the ecDNA, as GRO-seq and Ribo-Zero read densities on chromosomes were comparable between COLO320DM and COLO320HSR (Extended Data Fig. 2). We performed de novo transcript identification within the amplicon intervals using GRO-seq data and compared the same regions in COLO320DM versus COLO320HSR. We observed increases in both nascent and accumulated transcripts in COLO320DM compared to COLO320HSR, confirming that the increased transcription from ecDNA is amplicon-wide and not driven by a small number of differentially expressed transcripts (Fig. 1d). ecDNA-transcribed regions, including those not previously annotated, were also marked by the H3K36me3 histone mark, which is associated with RNA polymerase II elongation, providing orthogonal validation of rampant transcription (Fig. 1c,e and Extended Data Fig. 3a).

Elevated transcription is associated with single-stranded DNA (ssDNA) accumulation, due to the process of transcription itself, R loop formation from RNA:DNA hybrids and transcription–replication conflict^16^. To assess the influence of pervasive transcription on ecDNA structure, we performed kethoxal-assisted ssDNA sequencing (KAS-seq)^17,18^ to map ssDNA genome-wide. After normalizing to input to account for copy number differences, we observed a 1.4-fold increase in KAS-seq read density within the ecDNA amplicon in COLO320DM compared to COLO320HSR (Fig. 1b,c). The ssDNA regions on ecDNA extend from hundreds to over 20,000 basepairs (bp), and the majority of KAS-seq peaks overlap with transcribed regions, such as annotated non-coding transcripts (long non-coding RNAs, microRNAs, 60%) and novel transcripts identified in GRO-seq (18%; Fig. 1d,f and Extended Data Fig. 3b). Taken together, these results suggest that ecDNAs provide a permissive chromatin environment for pervasive transcription initiation, leading to accumulated RNA species and ssDNA.

## Transcription-driven RS on ecDNA

**Fig. 2:**
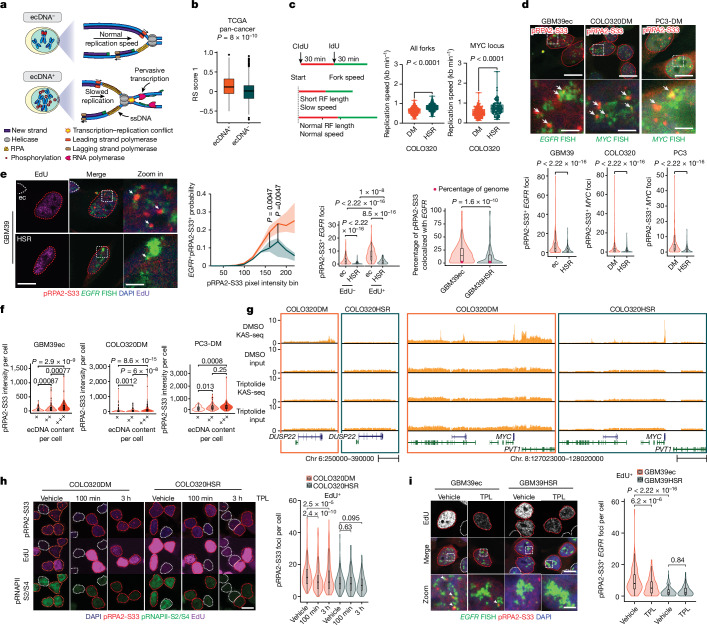
Transcription–replication conflict creates RS on ecDNAs. **a**, Schematics depicting transcription–replication conflict and RS. **b**, RS score 1 computed in TCGA patients grouped by ecDNA amplification status (*n*: 232, 582). **c**, DNA-fibre assay combined with *MYC* FISH in COLO320DM and COLO320HSR cells. Replication fork (RF) progression rate was measured globally (middle) or at the *MYC* locus (right) (box whiskers indicate min. to max.; *n*: 348, 317, 143, 101). **d**, Replication protein A phosphorylation: pRPA2-S33 IF combined with *EGFR* or *MYC* DNA FISH to show higher RS on ecDNA (*n*: 370, 274, 939, 568, 209, 244). **e**, pRPA2-S33 IF combined with *EGFR* FISH with 5-ethynyl-2′-deoxyuridine (EdU) added for 30 min; nuclei were co-stained by DAPI. Left, representative images. Second left, proportion of pixels with colocalization within each pRPA2-S33 pixel intensity bin (shade indicates median ± 25% quantile range, *n*: 10, 6). Second right, colocalization foci number (*n*: 267, 194, 104, 75). Right, percentage of pRPA2-S33 colocalized with *EGFR *(*n*: 371, 269). Red dot indicates percent of genome taken up by amplicon as calculated by WGS counts. **f**, Comparison of RS in tumour cells with different ecDNA content grouped by total DNA FISH intensity (GBM39ec,* n*: 111, 148, 111; COLO320DM, *n*: 282, 375, 282; PC3: 63, 83, 63). **g**, Genome tracks highlighting two regions within the ecDNA interval in COLO320 cells treated with triptolide or vehicle. **h**,**i**, Triptolide (TPL) treatment decreased RS on ecDNA in COLO320 (**h**) and GBM39 (**i**) cells (COLO320DM/HSR cells, *n*: 354, 350, 269, 130, 185, 161; GBM39ec/HSR cells, *n*: 139, 191, 264, 222). Boxplots **b**–**i** indicate centre line, median; limits, 25–75 quartiles; whiskers, 1.5× interquartile range or as otherwise specified. **d**–**f**,**h**–**i** were presented as violin plot and boxplot. Violin plot outlines kernel probability density. *P* determined by two-sided Wilcoxon test except unpaired Kolmogorov–Smirnov test in Fig. 2c. Scale bar, 10 µm (**d** (top row), **e** (left), **h**, **i** (top)), 2 µm (**d** (bottom row), **e** (right), **i** (bottom)), 50 kb (**g** (left)), 200 kb (**g** (right)).

**Extended Data Fig. 4:**
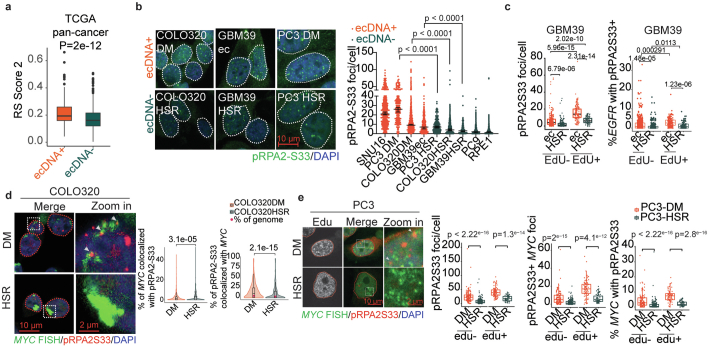
RS on ecDNA with different amplification sequence in different tumour cells. (**a**) RS score 2 computed in TCGA patients grouped by ecDNA amplification status (232 ecDNA positive, 582 ecDNA negative, two-sided Wilcoxon test. Boxplot parameter same as in Fig. 2b). (**b**) Left: Representative images of pRPA2-S33 immunofluorescence staining in 3 isogenic tumour cell line pairs. Right, pRPA2-S33 foci number in individual cells, each dot indicated one cell. (Median with 95% CI, ordinary one-way ANOVA with multiple comparisons test, n, 666, 205, 1191, 369, 244, 655, 244, 1505, 775). (**c**) Quantification of images in Fig. 2e, left: total pRPA2-S33 foci/cell; right: % of *EGFR* co-localized with pRPA2-S33. (Box plot parameters same as in Fig. 2b, Two-tailed Wilcoxon test, EdU- group: GBM39ec, n = 244, GBM39HSR, n = 143; EdU+ group: GBM39ec, n = 104, GBM39HSR, n = 72). (**d**) pRPA2-S33 immunofluorescence combined with *MYC* FISH staining in COLO320DM and COLO320HSR cells. Left: representative images. Middle: % of *MYC* colocalized with pRPA2-S33. Right: % of pRPA2-S33 that colocalized with *MYC*, red dot indicated % of genome that taken by amplicon as calculated by WGS counts in each cell line. (Data was presented as violin plot with the addition of boxplot. Violin plot outlines kernel probability density and boxplot parameters same as in Fig. 2b, Two-tailed Wilcoxon test COLO320DM, n = 939; COLO320HSR, n = 568). (**e**) pRPA2-S33 immunofluorescence combined with *MYC* FISH staining in PC3-DM and PC3-HSR cells, with EdU added 30 min before fixation. Left: representative images. 2nd left: pRPA2-S33 foci number. 2nd right: colocalized foci number between pRPA2-S33 and *MYC* FISH. Right: percentage of *MYC* co-localized with pRPA2-S33. (Box plot parameters were the same as in Fig. 2b, two-tailed Wilcoxon test, EdU+ group: PC3-DM, n = 81, PC3-HSR, n = 58; EdU- group: PC3-DM, n = 128, PC3-HSR, n = 184). (Scale bar represents 10 µm or as otherwise specified).

Pervasive transcription on ecDNA increases the possibility of transcription–replication conflict. When RNA polymerase II collides with the DNA replication machinery, progression of the replication fork is stalled, incorporation of new nucleotides is slowed, ssDNA behind the replication fork is exposed and bound by phosphorylated RPA2 protein (pRPA2-S33) and the cell experiences replication stress (RS)^19^ (Fig. 2a). This hypothesis predicts that ecDNA-containing cancer cells should have elevated DNA RS, and that the RS will be relieved by limiting transcription. First examining ecDNA-containing primary tumours, we grouped tumours from The Cancer Genome Atlas (TCGA) tumour patients into ecDNA-positive versus ecDNA-negative cohorts based on WGS data analysed by AmpliconArchitect^1^. We computed the RS score through two gene expression signatures identified in ref. ^20^ (RS score 1) and ref. ^21^ (RS score 2), and found a significantly higher RS score in ecDNA-containing tumours using both methods (Fig. 2b and Extended Data Fig. 4a). This result indicates that increased RS may be a common feature shared by ecDNA^+^ cancers. Next, conflicts between transcriptional and replicative machinery should lead to slower replication fork progression. We combined a DNA-fibre assay with DNA fluorescence in situ hybridization (FISH) to analyse replication fork dynamics in *MYC-*amplified isogenic COLO320DM versus COLO320HSR cells. Nascent DNA synthesis was labelled by sequential incubations with thymidine analogues IdU and CIdU. The velocity of the replication fork was then calculated by the length of each IdU/CIdU track. We observed a slower replication fork progression rate in COLO320DM compared with COLO320HSR cells; importantly, double labelling of thymidine analogue incorporation and *MYC* DNA FISH showed that ecDNA had significantly slower replication fork progression compared to the same sequence on the chromosome (Fig. 2c).

To directly visualize RS in individual tumour cells and to determine its subnuclear localization, we used immunofluorescence (IF) to detect RPA2 protein phosphorylation on serine 33 (pRPA2-S33), a marker of RS. We analysed pRPA2-S33 in a panel of cell lines, including three near-isogenic cell line pairs: COLO320DM/COLO320HSR (*MYC*-amplified colorectal cancer), GBM39ec/GBM39HSR (ref. ^2^) (*EGFR*-amplified glioblastoma) and PC3-DM/PC3-HSR (*MYC*-amplified prostate cancer) (Extended Data Fig. 1a–h), along with several other cell lines with or without ecDNA. Within each isogenic cell line pair, the amplified oncogene is shared but differs in its location on ecDNA or on a chromosome/HSR. We detected 2- to 3-fold higher pRPA2-S33 foci in ecDNA^+^ compared with ecDNA^−^ tumour cells, indicating increased RS in ecDNA-containing tumour cells (Extended Data Fig. 4b).

To determine whether RS is preferentially elevated on ecDNA, we performed concurrent DNA FISH to detect the ecDNA-amplified oncogene (*EGFR*) and IF to detect RS (pRPA2-S33) in GBM39ec cells. We added EdU labelling to detect actively replicating cells. We also examined these features in the isogenic counterpart, GBM39HSR, in which amplified *EGFR* has a similar copy number on chromosomes (Extended Data Fig. 1d)^2,11^. As hypothesized, we detected significantly higher RS on ecDNA in GBM39ec tumour cells, as measured by colocalization of pRPA2-S33 and *EGFR* FISH signal compared to GBM39HSR tumour cells, especially in EdU-positive cells. Notably, in pixels with increasing pRPA2-S33 intensity, a higher colocalization ratio was observed with a total of more than a 3-fold higher ratio on ecDNA as opposed to HSR, which suggests specific molecular interactions rather than just spatial organization differences between oncogenes on ecDNA and HSR (Fig. 2e). We continued to observe an increased pRPA2-S33 signal on *EGFR* in ecDNA^+^ cells after accounting for the total *EGFR* FISH signal, confirming that the higher RS on ecDNA compared to chromosomal amplification is not driven by differences in copy number (Extended Data Fig. 4c). To establish whether ecDNAs experience higher RS than the rest of the genome, we quantified the percentage of total nuclear pRPA2-S33 signal colocalized with *EGFR* in GBM39ec and GBM39HSR tumour cells. Based on WGS, ecDNA accounts for approximately 2% of the genomic content of GBM39ec cells. Therefore, if ecDNAs experienced a comparable level of RS compared to the rest of the genome, we would expect that they would account for a similar proportion of the total nuclear pRPA2-S33 signal. However, we found that a median of 14.5% of the total nuclear pRPA2-S33 signal is found on ecDNA, a 7-fold enrichment. In contrast, the proportion of pRPA2-S33 colocalized with *EGFR* is comparable to the relative genomic content of the amplicon in GBM39HSR (Fig. 2e). These findings indicate that RS is preferentially increased on ecDNA compared to the rest of the genome. Moreover, pRPA2-S33 IF combined with DNA FISH staining in two other near-isogenic cell line pairs containing *MYC* amplifications, COLO320 and PC3, also showed higher RS on ecDNA compared with HSR (Fig. 2d and Extended Data Fig. 4c–e). To confirm that ecDNAs are, in fact, drivers of RS, we binned individual cells by ecDNA copy number based on oncogene FISH intensity and compared the intensity of pRPA2S33 staining in cells with the highest 30%, lowest 30% and middle 40% of oncogene copy number in COLO320DM, GBM39ec and PC3-DM cells. We found that in all three cell lines, cells with the top 30% of ecDNA content have significantly higher RS than those with the bottom 30% of ecDNA content (Fig. 2f). Our results across multiple cancer cell types agnostic to the identity of the amplified oncogene collectively suggest that higher RS is a common feature of ecDNAs (Extended Data Fig. 4b–e).

**Extended Data Fig. 5:**
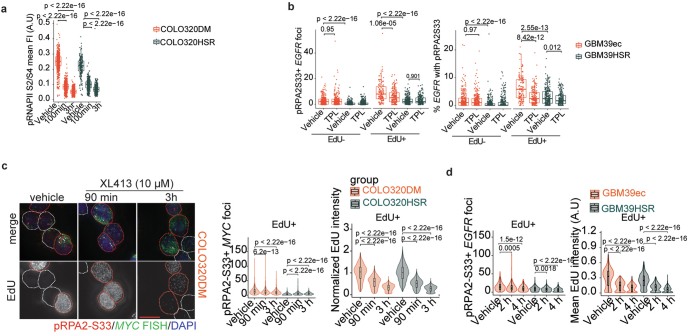
Transcription replication conflict drives replication stress in ecDNA containing tumour cells. (**a**) Mean pRNAPolII S2/S4 fluorescence intensity (arbitrary units) was measured in datasets shown in Fig. 2h. (Box plot parameters were the same as in Fig. 2b, two-tailed Wilcoxon test, n: 354, 350, 269, 130, 185, 161). (**b**) Quantification of dataset shown in Fig. 2i. left, pRPA2-S33 and *EGFR* colocalized foci number; right, percentage of *EGFR* colocalized with pRPA2-S33. (Box plot parameters were the same as in Fig. 2b, two-tailed Wilcoxon test, sample size from left to right: n = 280, 256, 332, 348, 138, 189, 253, 208) (**c**) pRPA2-S33 IF combined with *MYC* FISH staining in COLO320DM and COLO320HSR cells treated with XL413 (10 µM) for indicated time, EdU was added 30 min before fixing. Left, representative images in COLO320DM cells, middle, quantification of pRPA2-S33 and *MYC* DNA FISH co-localized foci in EdU+ cells; right, Mean EdU intensity normalized to vehicle treated cells in each group. (Two-tailed Wilcoxon test, sample number from left to right: 1835, 1241, 1195, 2578, 990, 1068). (**d**) pRPA2-S33 IF combined with EGFR FISH staining in GBM39ec and GBM39HSR cells treated with XL413 (10 µM) for indicated time, EdU was added 30 min before fixing. Left, quantification of pRPA2-S33 and *MYC* DNA FISH co-localized foci in EdU+ cells; right, Mean EdU intensity (arbitrary units) in EdU+ cells. (Two-tailed Wilcoxon test, sample number from left to right: 431, 494, 603, 674, 820, 829). (plots c-d were presented as violin plot with the addition of boxplot. Violin plot outlines kernel probability density and boxplot parameters same as in Fig. 2b. Scale bar represents 10 µm or as otherwise specified).

**Extended Data Fig. 6:**
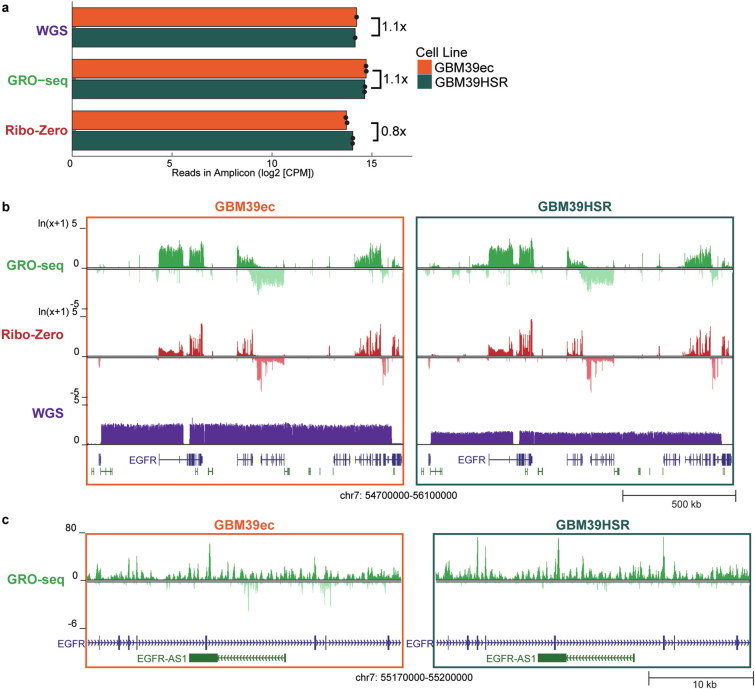
Transcription within the ecDNA interval of GBM39 cell lines. (**a**) Read density of genomic assays in GBM39ec and GBM39HSR within the ecDNA interval in total counts per million (CPM). The mean of two biological replicates is shown for GRO-seq, Ribo-Zero and KAS-seq; a single replicate is shown for WGS. (**b**) Genome tracks highlighting the GBM39 ecDNA interval. One representative biological replication for each condition is visualized. (**c**) Genome tracks highlighting the increased transcription at EGFR-AS1 in GBM39ec compared to GBM39HSR. One representative biological replication for each condition is visualized.

Having shown that ecDNAs have more open chromatin^11^, increased transcription and elevated RS, we set out to determine whether the elevated RS on ecDNA is a direct and potentially actionable consequence of pervasive transcription generated by ecDNA’s topology. We treated COLO320DM and COLO320HSR cells with triptolide, which inhibits transcription initiation through binding to the XPB subunit of the transcription factor complex TFIIH^22^. Active RNA polymerase II detected by IF showed that triptolide treatment significantly decreased transcriptional activity (Extended Data Fig. 5a). KAS-seq analysis in COLO320DM and COLO320HSR cells treated with triptolide revealed drastic reduction in ssDNA signals across the ecDNA amplicon (Fig. 2g). We found that triptolide treatment significantly decreased pRPA2-S33 foci in COLO320DM cells, with negligible effect in COLO320HSR cells (Fig. 2h), suggesting that transcription contributes to the elevated RS in COLO320DM cells. In the GBM39 isogenic model, although amplicon-wide nascent transcription is similar between ecDNA and HSR cells, we observed specific regions that are induced in GBM39ec compared to GBM39HSR, including the intragenic antisense transcript *EGFR-AS1* within the *EGFR* oncogene locus, resulting in convergent transcription (Extended Data Fig. 6a–c). Triptolide treatment of GBM39ec cells significantly decreased RS on ecDNA as detected by combined pRPA2-S33 IF and *EGFR* FISH, whereas no obvious difference was observed on HSR (Fig. 2i and Extended Data Fig. 5b). Furthermore, triptolide treatment only reduced the pRPA2S33 signal in actively replicating Edu^+^ GBM39ec cells (Extended Data Fig. 5b). Taken together, our results demonstrate that ecDNAs exhibit higher levels of RS than chromosomal loci, and that this increased RS is driven in large part by concurrent transcription and replication (Extended Data Fig. 5c,d).

## RS induces DNA damage on ecDNA

**Fig. 3:**
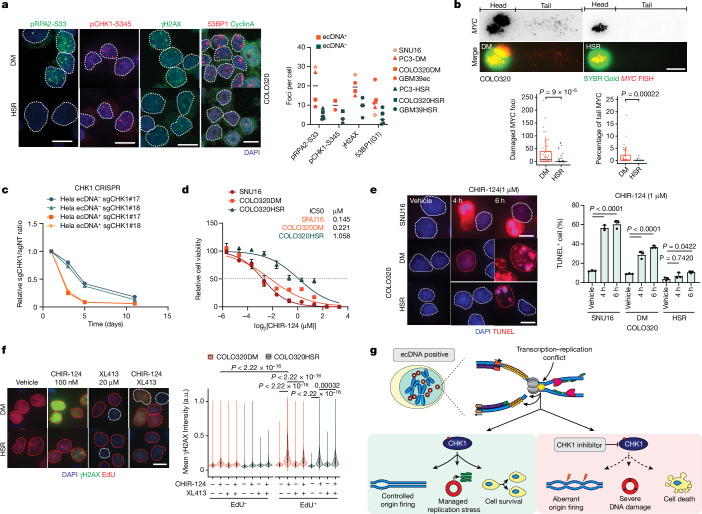
RS activated S-phase checkpoint and generated vulnerability to CHK1 inhibition in ecDNA-containing tumour cells. **a**, Detection of pRPA2-S33, γH2AX, pCHK1-S345 and 53BP1/cyclin A in multiple cancer cell lines with different ecDNA amplification status. Left, representative images in COLO320DM and COLO320HSR cells. Right, mean foci number in individual cell lines. Line indicates median; every dot indicates mean foci number in each cell line. **b**, Comet-FISH assay in COLO320DM and COLO320HSR cells. Top, representative images. Bottom left, *MYC* foci number in tail. Bottom right, percentage of *MYC* in comet tail (two-sided Wilcoxon test, *n*: 47, 60, 49, 33). **c**, Relative cell number of Hela ecDNA^+^ and Hela ecDNA^−^ cells transduced with sgRNAs targeting CHK1 normalized to cells transduced with non-targeted (NT) sgRNA over time. **d**, Cell viability curves of SNU16, COLO320DM and COLO320HSR in response to CHIR-124 for three days (*n* = 4, mean ± s.d.). **e**, TUNEL assay in cells subjected to CHIR-124 for indicated time (mean ± s.d., ordinary one-way ANOVA with multiple comparison test, *n* = 3). **f**, γH2AX IF in COLO320DM and COLO320HSR cells treated with CHIR-124 with or without the combination of CDC7i (XL413), with EdU added for 30 min. Left, representative images; red lines mark EdU^+^ and white lines mark EdU^−^ nuclei. Right, mean γH2AX intensity (arbitrary units). EdU^−^, *n*: 3,074, 4,246, 3,291, 4,742, 3,101, 3,770, 2,608, 2,091; EdU^+^, *n*: 2,428, 2,859, 2,909, 2,890, 3,346, 3,491, 3,232, 2,060; two-tailed Student’s *t*-test. **g**, Schematics depicting CHK1 activation in response to RS, which sensitizes ecDNA-containing tumour cells to targeted CHK1i through unscheduled replication origin firing and accumulation of excessive DNA damage, leading to cell death. Parameters for boxplots **b**,**f** and violin plot **f** are the same as Fig. 2 or as otherwise specified. Scale bar, 10 µm (**a**,**e**,**f**), 20 µm (**b**). a.u., arbitrary units.

**Extended Data Fig. 7:**
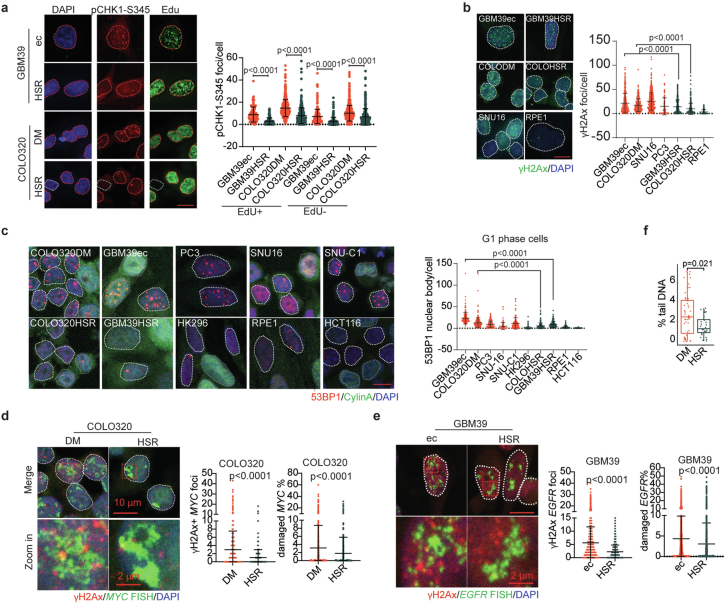
RS induces DNA lesions and activates S phase check point in ecDNA containing tumour cells. (**a**) pCHK1-S345 staining in 2 isogenic cell line pairs, with EdU added for 30 min. Left, representative images, red dotted lines mark EdU+ and white dotted lines mark EdU- nuclei; right, quantification of pCHK1 foci number. (mean± SD, two tailed Mann-Whitney test, n: 97, 192, 566, 339, 267, 252, 610). For COLO320HSR the same image is shown in Fig. 3a; they are used here to compare against multiple cell lines. (**b**) γH2AX staining in cell line panels with or without ecDNA. Left, representative image; right, quantification of γH2AX foci number per cell. (mean± SD, Ordinary one-way ANOVA with multiple comparison test, n: 402, 362, 499, 101, 388, 418, 80) (**c**) 53BP1 combined with CyclinA staining in cell line panels with or without ecDNA. Left, representative image. White dotted lines mark G1 phase cells (CyclinA-). Right, quantification of 53BP1 in G1 phase cells. (mean± SD, Ordinary one-way ANOVA with multiple comparison test, n, 180, 277, 240, 146, 126, 211, 188, 163, 221, 494). For COLO320DM and COLO320HSR the same images are shown in Fig. 3a; they are used here to compare against multiple cell lines. (**d**) γH2AX IF combined with *MYC* FISH staining in COLO320DM and COLO320HSR cells. Left: representative images. Middle, γH2AX and *MYC* colocalized foci number, right, percentage of *MYC* colocalized with γH2AX. (mean± SD, two-tailed Mann-Whitney test, sample size, n, 804, 411). (**e**) γH2AX IF combined with *EGFR* FISH staining in GBM39ec and GBM39HSR cells. Left, representative image. Middle, γH2AX and *EGFR* colocalized foci number, right, percentage of *EGFR* colocalized with γH2AX. (mean± SD, two tailed Mann-Whitney test, sample size, GBM39ec, n = 1638; GBM39HSR, n = 1863). (**f**) Quantification of percentage of tail DNA content with the dataset shown in Fig. 3b. (Box plot parameters were same as in Fig. 2b, two-tailed Wilcoxon test, COLO320DM, n = 49, COLO320HSR, n = 33). (Scale bar represents 10 µm or specified).

RS contributes to endogenous DNA damage because stalled replication forks are unstable and prone to breakage, generating DNA lesions^23^. Therefore, we hypothesized that ecDNA-containing tumour cells may have higher baseline levels of DNA damage. We tested this hypothesis using two markers for DNA damage: γH2AX marks all double-stranded DNA breaks and 53BP1 marks unrepaired DNA damage that arises from DNA replication during the previous cell cycle specifically in G1 daughter cells. In a panel of ecDNA^+^ and ecDNA^−^ cancer cell lines, including three near-isogenic cell line pairs, we found that in addition to having more pRPA2-S33 foci, ecDNA^+^ cells showed an average increased number of γH2AX and 53BP1 foci than the corresponding isogenic HSR and/or other ecDNA^−^ cell lines (Fig. 3a and Extended Data Fig. 7b,c). Combined γH2AX IF with DNA FISH staining in isogenic cell line pairs confirmed enhanced DNA damage on ecDNAs, compared to chromosomal amplicons (Extended Data Fig. 7d,e). To further confirm the presence of DNA damage on ecDNA itself, we performed an alkaline comet assay combined with *MYC* FISH staining in COLO320DM and COLO320HSR cells, where damaged DNA appears in the tail region of the comet. We observed significantly more *MYC* foci in the tail region of COLO320DM cells compared to COLO320HSR cells (Fig. 3b), which have a comparable amplicon copy number. These data demonstrate elevated DNA damage on ecDNAs, relative to the same loci amplified on chromosomes. Thus, ecDNA-containing cancer cells may be hyperreliant on the RS regulation machinery to cope with the elevated levels of baseline DNA damage driven by transcription–replication conflicts.

## ecDNA sensitizes cells to CHK1i

We reasoned that this hyperreliance on the RS regulation machinery in ecDNA-bearing tumour cells might generate an actionable therapeutic vulnerability. To cope with stalled replication forks, cells employ a signalling cascade known as the S-phase checkpoint to ensure that they do not progress to mitosis when the DNA is incompletely replicated. Checkpoint kinase 1 (CHK1), which is phosphorylated when the checkpoint is activated, is a central node for this checkpoint pathway. We detected more pCHK1-S345 by IF in ecDNA-containing tumour cells compared with the corresponding isogenic HSR cells (Fig. 3a and Extended Data Fig. 7a), indicating that transcription–replication conflict on ecDNA leads to S-phase checkpoint activation in ecDNA-containing tumour cells. In the absence of a functioning checkpoint, cells with highly damaged DNA proceed through the cell cycle, leading to cell death^24^. We therefore hypothesized that ecDNA-containing tumour cells, due to their intrinsic heightened RS, would be hyperreliant on CHK1 to manage DNA damage, and that CHK1 inhibition (CHK1i) could trigger preferential cell death in ecDNA-containing tumour cells.

**Extended Data Fig. 8:**
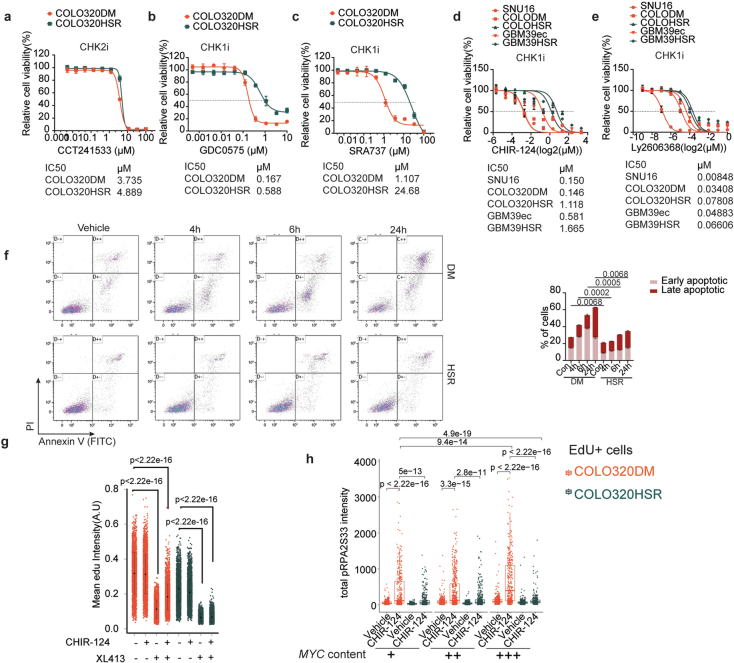
ecDNA containing tumour cells are sensitive to targeted CHK1 inhibition. (**a**-**e**) Cell viability curve of COLO320DM, COLO320HSR, GBM39ec, GBM39HSR and SNU16 in response to different chemicals targeting CHK1 or CHK2. a. CHK2i, CCT241533; b. CHK1i, GDC0575; c. CHK1i, SRA737; d, CHK1i, CHIR-124; e, CHK1i, Ly2606368. Half maximal inhibitory concentrations (IC50) for each inhibitor in individual cell lines were listed on the bottom. (sample size in a-c, n = 2; d-e, n = 4, mean ± SD) (**f**) FACS analysis of Annexin V staining in COLO320DM and COLO320HSR cells subjected to 1 µM CHIR-124 for indicated time. Left, gating setting and representative plots, Early apoptotic cells: Annexin V + and PI-; late apoptotic cells: Annexin V + and PI +. Right, % of apoptotic cells (mean± SEM, P values quantified by two-tailed students’ t test, n = 2.) (**g**) Quantification of mean EdU intensity (arbitrary units) in dataset shown in Fig. 3f. (mean± SD, P values quantified by two-tailed students’ t test. Sample size from left to right: n = 419, 284, 1085, 596, 209, 242). (**h**) pRPA2-S33 IF combined with *MYC* FISH staining in COLO320DM and COLO320HSR cells treated with 100 nM CHIR-124 for 2 h, EdU was added 30 min before fixing. Accumulation of further RS upon CHK1i was quantified by total pRPA2-S33 fluorescence intensity in EdU+ cells. COLO320DM and COLO320HSR cells were grouped into 3 subgroups with different amplicon content based on *MYC* DNA FISH staining. (Box plot parameters were same as in Fig. 2b, two-tailed Wilcoxon test, Two-tailed Wilcoxon test, sample number from left to right: 355, 337, 466, 315; 472, 448, 622, 420; 354, 337, 466, 315).

To test this hypothesis, we used clustered regularly interspaced short palindromic repeats (CRISPR) to knock out the gene encoding CHK1 in a pair of Hela cell lines with or without *DHFR* amplification on ecDNA. Two different single guide RNAs (sgRNAs) targeting CHK1 induced 2- to 3-fold higher growth inhibition in ecDNA^+^ compared with ecDNA^−^ Hela cells across different time points (Fig. 3c). We next inhibited CHK1 pharmacologically using CHIR-124 (ref. ^25^) and found that ecDNA-containing tumour cells were more sensitive to CHK1i than their corresponding isogenic HSR cells, with a half-maximal inhibitory concentration (IC_50_) approximately 4-fold higher in COLO320HSR compared to COLO320DM cells (Fig. 3d). The susceptibility of ecDNA-containing tumour cells to CHK1 inhibition was confirmed with three structurally different CHK1 inhibitors—GDC-0575, SRA737 and CHIR-124—whereas the checkpoint kinase 2 (CHK2) inhibitor CCT241533 showed no differential inhibitory effect between ecDNA^+^ and ecDNA^−^ isogenic cell lines (Extended Data Fig. 8a–e). More importantly, suppression of cell growth by CHK1i was mediated through induction of cell death, as a more rapid and higher degree of cell apoptosis was observed in ecDNA-containing tumour cells treated with CHIR-124, as detected by terminal deoxynucleotidyl transferase dUTP nick end labelling (TUNEL; Fig. 3e) and PI-Annexin V staining (Extended Data Fig. 8f).

As a master effector of S-phase checkpoint, CHK1 activation maintains cell viability by restricting cell cycle progression^24,26^, limiting late replication origin firing to prevent excessive DNA damage accumulation, and protecting stalled replication forks^27,28^. γH2AX IF combined with EdU labelling in COLO320DM and COLO320HSR cells treated with CHIR-124 showed that CHK1i induced significantly higher DNA damage in COLO320DM compared with COLO320HSR cells, especially in S-phase cells as indicated by EdU^+^ staining, consistent with the function of CHK1 in replication (Fig. 3f). Furthermore, inhibition of replication origin firing by CDC7i (XL413), indicated by the decreased EdU^−^ staining intensity (Extended Data Fig. 8g), partially blocked DNA damage induced by CHK1i (Fig. 3f), suggesting that CHK1i leads to extensive RS and DNA damage partially through unscheduled replication origin firing. Furthermore, by combining pRPA2-S33 IF with *MYC* FISH, we found that the increased sensitivity of COLO320DM cells to CHK1i was consistent, regardless of ecDNA copy number (Extended Data Fig. 8h).

Taken together, our findings demonstrate that transcription–replication conflict, RS and increased baseline DNA damage are common features of ecDNAs and drive activation of the S-phase checkpoint. Targeted CHK1i in ecDNA^+^ cells leads to unscheduled replication origin firing and accumulation of DNA damage. Furthermore, the high levels of transcription–replication conflict and RS drive a selective vulnerability to CHK1i in ecDNA^+^ cells compared to ecDNA^−^ cells, raising the possibility for an effective ecDNA-directed therapy (Fig. 3g).

## Oral CHK1i stops ecDNA^+^ tumours

**Fig. 4:**
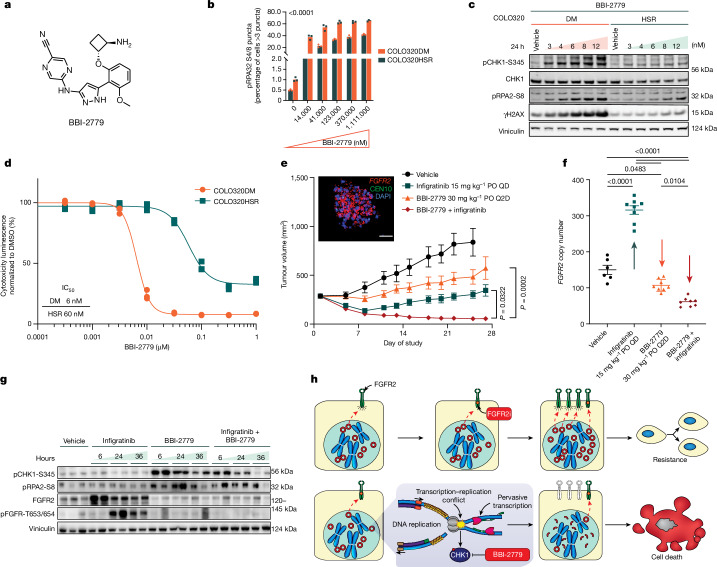
Oral CHK1i in combination with a pan-FGFRi demonstrates synergistic antitumour activity and inhibits acquired resistance to targeted therapy manifested by ecDNA. **a**, Chemical structure of BBI-2779. **b**,**c**, Dose-dependent induction of RS and associated biomarkers measured by phosphorylated RPA32 Ser8 level using IF (**b**) and immunoblotting (**c**). For **b**, significance determined using ordinary two-way ANOVA, *n* = 3. **d**, Differential tumour cell antiproliferation activity of BBI-2779 in COLO320DM and HSR cells (*n* = 3). **e**, Embedded FISH image of SNU16 cells demonstrating *FGFR2*^+^ ecDNA. SNU16 cells were grown as tumour xenografts in mice. After tumour establishment (approximately 285 mm^3^), mice were treated with vehicle, BBI-2779 (30 mg kg^−1^), infigratinib (15 mg kg^−1^) or BBI-2779 (30 mg kg^−1^) plus infigratinib (15 mg kg^−1^) for 25 days (vehicle) or 27 days (other arms). Mean tumour volumes ± s.e.m. are shown (*n* = 8 mice per group). **f**, *FGFR2* copy number was evaluated by quantitative polymerase chain reaction (qPCR) on tumour DNA. Significance was determined by one-way ANOVA with Tukey’s multiple comparisons. **g**, Immunoblots of tumour lysates measuring elevated RS, DNA damage and abrogation of oncoprotein FGFR2 expression (*n* = 3/8 mice per group). **h**, ecDNA-amplified oncogenes are hypertranscribed, resulting in elevated RS and reliance on CHK1 to manage DNA replication to maintain oncoprotein overexpression and proliferation. CHK1i results in uncontrolled origin firing and failed cell cycle checkpoints, exacerbating RS in ecDNA-enabled tumour cells. Synthetic lethality to CHK1i in ecDNA^+^-oncogene-amplified tumour cells is synergistic with targeted therapy resulting in enhanced cytotoxicity. Scale bar, 10 µm. PO, oral; QD, once-daily; Q2D, every other day. Source Data

**Extended Data Fig. 9:**
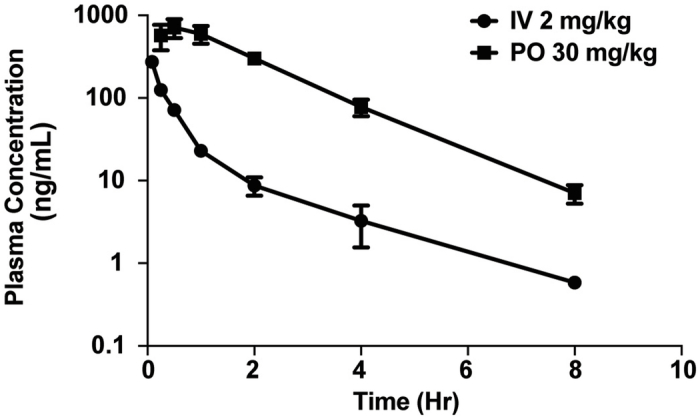
BBI-2779 has optimal PK exposure in mice. Plasma concentration time curve of BBI-2779 in mouse administered either intravenously (IV) at 2 mg kg^−^^1^ or orally (PO) at 30 mg/kg^−^^1^. Data are mean ± s.e.m., n = 3 per group. Source Data

Despite convincing preclinical data and preliminary evidence of single-agent clinical activity for CHK1i, there are currently no approved CHK1 inhibitors for any cancer indication. Several limitations of prior CHK1 inhibitors include insufficient potency, potential off-target liabilities (for example, CHK2), and overlapping toxicity in combination with DNA-damaging chemotherapy^29^. To further interrogate the potential of CHK1i as a treatment for ecDNA^+^ cancers, we advanced BBI-2779, an orally bioavailable, potent and selective small molecule inhibitor of CHK1 (Fig. 4a). The potency of BBI-2779 against CHK1 was confirmed in vitro using biochemical enzyme inhibition and cellular biomarker assays. The biochemical inhibition IC_50_ of BBI-2779 against CHK1 was found to be 0.3 nM, while cellular induction of RS (as judged by pCHK1-S345, due to CHK1 phosphorylation by upstream kinases) in tumour cells was observed to be 3 nM. BBI-2779 has superior biochemical and selective cell growth inhibition compared to other orally bioavailable CHK1 inhibitors tested (IC_50_ of ecDNA^+^ CellTiter-Glo proliferation is around 18–168-fold more potent) (Table 1). The inhibitor was observed to be greater than 160-fold selective for CHK1 over CHK2, suggestive of high pharmacological specificity (Table 1). BBI-2779 also displays excellent bioavailability (%*F* = 71) and good exposure in rodents, allowing for robust CHK1 target coverage after oral administration (Table 2 and Extended Data Fig. 9).

**Table 1:** In vitro and cellular potency of BBI-2779 and of reference compounds

	BBI-2779	GDC-0575	SRA737
CHK1 biochemical potency (IC_50_), nM	0.3	12	85
CHK1/CHK2 selectivity	160×	1.5×	2,000×
CHK1 cellular potency AlphaLisa (IC_50_), nM	3	122	1,500
CTG proliferation ecDNA^+^ (IC_50_), nM	6	105	1,010

AlphaLISA pCHK1-S345 activity was assessed in HT29 cells, while antiproliferation potency was evaluated in COLO320DM cells.

**Table 2:** Pharmacokinetic parameters of BBI-2779 indicate that it is well tolerated in mice

Pharmacokinetic parameter	BBI-2779
In vivo cl (ml min^−^^1^ kg^−^^1^)	229
*t*_1/2_ (h)	1.11
*t*_max_ (h)	0.5
*C*_max_ (ng ml^–1^)	713
AUC_inf_ (h ng ml^–1^)	1,568
%*F*	72

Oral bioavailability was determined in fasted male CD-1 mice dosed at 30 mg kg^−^^1^ (*n* = 3). AUC_inf_, area under concentration-time curve; cl, plasma clearance; *C*_max_, maximum observed concentration; %*F*, % oral bioavailability; *t*_1/2_, terminal half life; *t*_max_, time maximum concentration.

As ecDNA^+^-oncogene-amplified tumour cells harbour elevated intrinsic RS and are sensitive to other CHK1 inhibitors (Fig. 3d), we hypothesized that they would also be hypersensitive to BBI-2279. Consistent with this notion, BBI-2779 treatment of COLO320DM cells resulted in a significantly greater dose-dependent increase in the expression of the RS biomarker pRPA2-S8 compared to COLO320HSR (Fig. 4b). COLO320DM cells also showed a greater dose-dependent increase of pCHK1-S345 and γH2AX, as determined by Western blotting (Fig. 4c). The concentration-dependent induction of RS induced by BBI-2779 directly correlated with enhanced cytotoxicity in the COLO320DM cells as compared to COLO320HSR cells, with an approximately 10-fold difference in IC_50_ between COLO320DM and COLO320HSR cells, demonstrating synthetic lethality in the ecDNA^+^ context (Fig. 4d).

**Extended Data Fig. 10:**
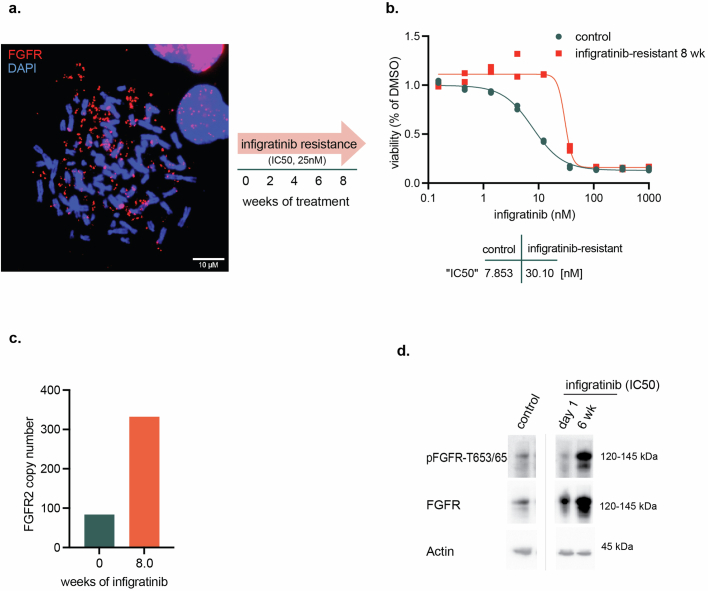
Targeted therapeutic resistance shaped by intracellular ecDNA -driven oncogene amplification. (**a**) *FGFR2* (red) FISH imaging of cells in metaphase demonstrate amplification of *FGFR2* oncogene on ecDNA in SNU16 cells. Nuclear staining is illustrated using DAPI (blue). Representative FISH image from multiple independent cytogenetic analysis with similar results. Scale bar represents 10 µm. (**b**) Timeline of experimental overview. After 8 weeks of infigratinib treatment (EC50 dose of 25 nM), cells were assessed for infigratinib resistance. A 3-day Cell Titer Glo reveals resistance in SNU16 cells treated with infigratinib (n = 2 biologically independent samples). (**c**) qPCR based quantification of *FGFR2* oncogene numbers after 8 weeks of infigratinib treatment showing SNU16 cells resistant to infigratinib with significant increase in *FGFR2* target selection/amplification (qPCR data represents n = 1 biological sample. Multiple repeat analysis reveal similar results). (**d**) Western blotting illustrating enhanced expression of FGFR signaling pathways involved in therapeutic resistance (Protein data represents n = 1 biological sample. Multiple repeat analysis reveal similar results). Lane 1 (control) is from a non-contiguous portion of the gel from lanes 2 and 3 (infigratinib treated samples). Source Data

Applying targeted therapy pressure to the protein products of oncogenes amplified on ecDNA induces cancer cells to evade such pressures, either by increasing ecDNA amplification of the dominant oncodriver (Extended Data Fig. 10a–d), or by ecDNA amplification of a new bypass oncogene^30^. We therefore investigated whether combining targeted therapy with CHK1i in ecDNA-amplified tumour cells provides a synergistic therapeutic effect resulting in cancer cell death and tumour regression. The synergistic antitumour activity and pharmacodynamics of BBI-2779 was evaluated in combination with the pan-FGFR tyrosine kinase inhibitor infigratinib in the *FGFR2-*amplified ecDNA^+^ gastric cancer SNU16 xenograft tumour model.

Single-agent BBI-2779 or infigratinib resulted in significant tumour growth delay with mean per cent tumour growth inhibition of 64% and 97% compared to the vehicle arm on day 25 (*P* < 0.05 and *P* < 0.0005, respectively) (Fig. 4e). Prolonged treatment of SNU16 tumour cells in vitro and SNU16 xenograft tumours in vivo with infigratinib resulted in tumour cell stasis for a period of 1–2 weeks, followed by acquired resistance to infigratinib and reinitiation of tumour growth concomitant with increased *FGFR2* amplifications on ecDNA (Fig. 4f and Extended Data Fig. 10a–c). The lack of robust or sustained antitumour activity observed with infigratinib alone is consistent with the absence of compelling clinical efficacy reported for pan-FGFR inhibitors in FGFR1/2/3-amplified settings^31^. Increased *FGFR2* gene amplification correlated with FGFR2 protein levels that likely out-titrate the exposure of infigratinib at its maximally tolerated dose in mice (Fig. 4g and Extended Data Fig. 10d). The combination of BBI-2779 plus infigratinib resulted in significant tumour growth inhibition compared to vehicle-treated animals (*P* < 0.0001), with tumour regressions observed over the duration of the study, which was directly correlated with the suppression of further (adaptive) *FGFR2* oncogene copy number amplification on ecDNA, otherwise induced by single-agent infigratinib (Fig. 4e,f). As expected, both single-agent BBI-2779 and combination of BBI-2779 plus infigratinib treatment resulted in a heightened tumour expression of RS biomarkers pCHK1-S345 and pRPA2-S8 compared to vehicle-treated tumours (Fig. 4g). Taken together, these findings demonstrate synergistic antitumour activity by combining a selective CHK1 inhibitor with a targeted therapy against the protein product of the amplified driver oncogene to attenuate ecDNA-mediated resistance. Uncontrolled origin firing caused by selective CHK1i severely disrupts oncogene expression on hypertranscribed ecDNA templates, thereby rendering the oncogene-addicted tumour cells highly vulnerable to FGFR inhibition (Fig. 4h).

## Discussion

ecDNA is a pernicious driver of tumour evolution because it is a platform for massive oncogene expression and rapid genome adaptation. Here we show that the transcriptional advantage of ecDNA can be turned on its head to selectively target ecDNA-containing tumours. The increased transcription of ecDNA is not limited to the protein-coding oncogene loci, but also extends to multiple non-coding intergenic and antisense regions throughout ecDNAs, implying violation of evolved configurations of gene directionality and replication origins in the genome. The pervasive transcription initiation is consistent with increased chromatin accessibility and promiscuous enhancer–promoter contacts on ecDNA^9,10^. Thus, rampant ecDNA transcription comes at the cost of increased transcription–replication conflict that cancer cells must manage. DNA damage has been previously associated with ecDNA-containing cancers principally as a source of ecDNA generation^8^. Our results show that, once formed, ecDNAs themselves become a major driver of DNA damage. The RNA transcription and DNA replication machineries are two processive holoenzymes that both run along DNA; they must take turns or risk collision. Our findings demonstrate that concurrent transcription and replication on ecDNA drives a significant increase in DNA damage, and cancer cells become heavily reliant on the S-phase CHK1 to limit origin firing. The alternative for the cancer cell is to limit ecDNA transcription and lose oncogene overexpression, undermining their unique oncogenic and adaptive growth advantage. Differences in replication rate or origin firing at ecDNAs may also contribute to their increased RS, which should be addressed in future studies. Notably, the elevated levels of DNA damage on ecDNA due to RS may drive further evolution of the ecDNAs themselves. However, there are likely many mechanisms functioning in parallel that drive ecDNA mutations over time, including differential expression of some DNA damage repair pathways in ecDNA^+^ cancers^32^ and increased APOBEC3-mediated mutagenesis^33^. Our study examined a limited number of cell line models for mechanistic studies and potential indirect effects. As oncogene encoded on ecDNA (*MYC, EGFR*) drives transcription and cell replication, ecDNAs may promote transcription–replication conflicts indirectly throughout the genome. Nonetheless, both direct and indirect effects of ecDNA highlight transcription–replication conflict as a therapeutic opportunity in ecDNA^+^ cancers.

We tested the concept that enhancing transcription–replication conflict will cause ecDNA-containing tumour cells to self-destruct. Inhibition of CHK1 substantially increases ecDNA damage during DNA replication and leads to preferential killing of ecDNA-containing cancer cells. There are currently no approved CHK1 inhibitors for use in cancer patients. Despite convincing preclinical data and preliminary evidence of single-agent clinical activity for CHK1i, a predictive biomarker(s) and an optimal clinical development strategy have been lacking. Furthermore, a major challenge to the successful clinical development of CHK1 inhibitors has been the lack of reliable methods to identify high-RS tumours that are predicted to be hypersensitive to CHK1i^34–41^. Long durability of CHK1i in vivo is likely required to exploit unscheduled DNA replication to ensure cancer cell death. The results presented here suggest a promising strategy for a next-generation CHK1 inhibitor to target ecDNA-containing cancers. Notably, CHK1i showed synergy with a targeted therapy blocking the ecDNA oncogene-encoded protein product and prevented the adaptive elevation of ecDNA copy number that previously foiled single-agent therapies targeting oncogene-amplified protein products. Previous successes in cancer therapy have exploited the synthetic lethality of cancer-specific cellular deficiencies, for example PARP inhibition in BRCA2-deficient cancer cells^42^. Our work demonstrates the feasibility of a synthetic lethality of cancer-specific cellular excess to turn the molecular advantages of ecDNA in cancer against itself.

## Methods

### Antibodies and reagents

#### Antibodies

Antibodies were procured from the following: H3K36me3 (Abcam, catalogue no. ab9050), γH2AX (Millipore, catalogue no. 05-636 for IF), γH2AX (Cell Signaling Technology, catalogue no. CST9718 for western blot), pRPA2S33 (Novus Biological, catalogue no. NB100-544), pCHK1S345 (Invitrogen, catalogue no. PA5-34625), 53BP1 (Novus Biological, catalogue no. NB100-304), cyclin A (BD Biosciences, catalogue no. 611268), pRNAPII S2/S4 (Abcam, catalogue no. ab252855), pCHK1-S345 (Cell Signaling Technology, catalogue no. CST2348), CHK1 (Abcam, catalogue no. ab32531), pRPA32/RPA2-Ser8 (Cell Signaling Technology, catalogue no. 54762 S), Vinculin (Cell Signaling Technology, catalogue no. CST13901), pFGFR2-Tyr653/654 (Cell Signaling Technology, catalogue no. CST3476S) and FGFR2 (Cell Signaling Technology, catalogue no. CST11835S).

#### Chemicals

Chemicals were procured from the following: CHIR-124 (Selleckchem, catalogue no. S2683), XL413 (Selleckchem, catalogue no. S7547) and triptolide (Millipore, catalogue no. 645900-5MG).

### Cell culture

GBM39ec, GBM39HSR and HK296 were patient-derived neurosphere cell lines and were established as previously described^2,7^. The parental PC3 line was obtained from ATCC. PC3 DM and PC3 HSR lines were isolated by the Mischel Lab through single-cell expansions of the parental PC3 line and are available from the Mischel Lab upon request. All the other cell lines were purchased from ATCC. Human prostate cancer cell line PC3 DM, PC3 HSR; colorectal cancer cell line COLO320DM, COLO320HSR; gastric cancer cell line SNU16; lung cancer cell line PC9 and hTERT-immortalized retinal pigment epithelial cell line RPE1 were cultured in 4.5 g l^−^^1^ glucose-formulated Dulbecco’s Modified Eagle’s Medium (Corning) supplemented with 10% fetal bovine serum (FBS; Gibco). For GRO-seq and ChIP–seq, COLO320DM and COLO320HSR were grown in Roswell Park Memorial Institute 1640 with GlutaMAX (Gibco) with 10% FBS. GBM39ec, GBM39HSR and HK296 cell lines were cultured in Dulbecco’s Modified Eagle’s Medium/F12 (Gibco, catalogue no. 11320-033) supplemented with 1× B27 (Gibco, catalogue no. 17504-01), 20 ng ml^−^^1^ epidermal growth factor (Sigma, catalogue no. E9644), 20 ng ml^−^^1^ fibroblast growth factor (Peprotech, catalogue no. AF-100-18B), 1–5 µg ml^−^^1^ heparin (Sigma, catalogue no. H3149) and 1× GlutaMAX (Gibco, catalogue no. 35050-061). GBM39 cells used in sequencing assays were cultured without additional GlutaMAX. All the cells were maintained at 37 °C in a humidified incubator with 5% CO_2_. Cell lines routinely tested negative for mycoplasma contamination.

### GRO-seq

COLO320DM and COLO320HSR RNA was prepared by washing cells with ice-cold phosphate-buffered saline (PBS), then adding ice-cold LB (10 mM Tris-HCl pH 7.4, 2 mM MgCl2, 3 mM CaCl2, 0.5% IGEPAL-CA630, 10% glycerol, 1 mM DTT, protease inhibitors (Roche, catalogue no. 11836170001), RNase inhibitor (Ambion, catalogue no. AM2696)) and scraping cells into a 15 ml conical tube. Cells were spun at 1,000*g* for 10 min at 4 °C. Supernatant was removed and pellet was thoroughly resuspended in 1 ml LB using a wide bore tip. An additional 9 ml LB was added and then cells were spun at 1,000*g* for 10 min at 4 °C. Cells were resuspended in LB and spun down. Pellets were resuspended in ice-cold freezing buffer (50 mM Tris-HCl pH 8.3, 5 mM MgCl_2_, 40% glycerol, 0.1 mM EDTA, 0.2 μl RNase inhibitor per ml of freezing buffer) and spun at 2,000*g* for 2 min at 4 °C. Nuclei were resuspended in 100 μl freezing buffer per 5 million cells. A nuclear run-on master mixed was prepared (10 mM Tris-HCl pH 8.0, 5 mM MgCl2, 1 mM DTT, 300 mM KCl, 0.5 mM ATP, 0.5 mM GTP, 0.003 mM CTP (unlabelled ribonucleotide triphosphates from Roche, catalogue no. 11277057001), 0.5 mM Bromo-UTP (Sigma, catalogue no. B7166), 1% Na-laurylsarcosine, 1 μl RNase inhibitor per 100 μl) and preheated to 30 °C. An equal volume of master mix was added to aliquoted nuclei (5 million nuclei per replicate) and incubated at 30 °C for 5 min with gentle shaking. DNase digestion was performed using RQ1 DNase I and RQ1 buffer (Promega, catalogue no. M610A) for 30 min at 37 °C; the reaction was stopped with the addition of stop buffer to a final concentration of 10 mM Tris-HCl pH 7.4, 1% sodium dodecyl sulfate (SDS), 5 mM EDTA, 1 mg ml^−^^1^ proteinase K. Samples were incubated for 1 h at 55 °C. NaCl was added to final concentration of 225 mM. Two phenolchloroform extractions were done, followed by one extraction with chloroform. RNA was precipitated in 75% EtOH with 1 μl glycoblue (Ambion, catalogue no. 9516) overnight at −20 °C.

For GBM39ec and GBMHSR, cells were washed with ice-cold PBS and then spun for 5 min at 500*g* at 4 °C. Cells were then resuspended in ice-cold 10 ml swelling buffer (10 mM Tris-HCl pH 7.5, 2 mM MgCl2, 3 mM CaCl2, protease inhibitor, RNase inhibitor) and incubated on ice for 5 min. Cell were spun at 400*g* for 10 min at 4 °C and resuspended in 10 ml ice-cold glycerol swelling buffer (0.9× swelling buffer, 10% glycerol). While agitating the tube, 10 ml ice-cold lysis buffer (glycerol swelling buffer, 1% IGEPAL-CA630) was slowly added. Samples were incubated on ice for 5 min, then another 25 ml lysis buffer was added and samples were spun for 5 min at 600*g* at 4 °C. Samples were resuspended in ice-cold freezing buffer (50 mM Tris-HCl pH 8.0, 5 mM MgCl2, 40% glycerol, 0.1 mM EDTA, RNase inhibitor) and spun at 900*g* for 6 min at 4 °C. An equal volume of pre-warmed nuclear run-on master mix was added to aliquoted nuclei (10 million nuclei per replicate) and incubated at 30 °C for 7 min with gentle shaking. Samples were then mixed thoroughly with 600 μl Trizol LS and incubated at room temperature for 5 min. Next, 160 μl chloroform was added to each sample, shaken vigorously, then incubated at room temperature for 3 min and centrifuged at 12,000*g* at 4 °C for 30 min. NaCl was added to the aqueous phase to a final concentration of 300 mM and RNA was precipitated in 75% EtOH with 1 μl glycoblue overnight at −20 °C.

For all cell types, after overnight RNA precipitation, RNA was spun for 20 min at 21,130*g* at 4 °C. RNA pellets were washed in fresh 75% EtOH, briefly air-dried and then resuspended in 20 μl water. Base hydrolysis was performed using 5 μl 1 N NaOH for 10 min and then neutralized with 25 μl 1 M Tris-HCl pH 6.8. Buffer exchange was performed using P30 Micro columns (Bio-Rad, catalogue no. 7326250), then treated with RQ1 DNase I and RQ1 buffer and incubated at 37 °C (10 min for COLO320 and 30 min for GBM39). Buffer exchange was performed again. Samples were treated with 3 μl T4 polynucleotide kinase (PNK; New England Biolabs, catalogue no. M0201), 1× PNK buffer, 2 μl 10 mM ATP and 2 μl RNase inhibitor and incubated for 1 h at 37 °C. Another 2 μl PNK was added per sample and incubation was continued for 30–60 min. RNA decapping was performed by adding ammonium chloride (final concentration 50 mM), poloaxamer 188 (final concentration 0.1%), 2 μl messenger RNA decapping enzyme (New England Biolabs, catalogue no. M0608S) and 1 μl RNase inhibitor and incubated at 37 °C for 30 min. EDTA was then added to the final concentration of 25 mM and samples were incubated at 75 °C for 5 min. Samples were then incubated on ice for at least 2 min. Sample volume was then brought to 100 μl with binding buffer (0.25× SSPE, 1 mM EDTA, 0.05% Tween 20, 37.5 mM NaCl, RNase inhibitor). During T4 PNK treatment, 60 μl anti-BrdU agarose beads (Santa Cruz Biotechnology, catalogue no. sc-32323ac) per sample were equilibrated in 500 μl binding buffer by rotating for 5 min at room temperature, spun and washed again in binding buffer. Beads were then blocked in blocking buffer (1× binding buffer, 0.1% polyvinylpyrrolidone, 1 ug ml^−^^1^ ultrapure bovine serum albumin (BSA), RNase inhibitor) by rotating for 1 h at room temperature. Beads were then washed twice in binding buffer and resuspended in 400 μl binding buffer. Decapped RNA was then added to the blocked beads and rotated for 1 h at room temperature. Beads were then washed once in binding buffer, once in low-salt buffer (0.2× SSPE, 1 mM EDTA, 0.05% Tween 20, RNase inhibitor), once in high-salt buffer (0.2× SSPE, 1 mM EDTA, 0.05% Tween 20, 137.5 mM NaCl, RNase inhibitor) with 3 min of rotation, and twice in Tris-EDTA-Tween20 buffer (10 mM Tris-HCl pH 8.0, 1 mM EDTA, 0.05% Tween 20, RNase inhibitor). All spins with agarose beads were performed for 2 min at 1000*g* at room temperature and all washes were performed in 500 μl buffer rotating for 5 min at room temperature unless otherwise noted. Samples were then eluted in elution buffer (50 mM Tris-HCl pH 7.5, 20 mM DTT, 1 mM EDTA, 150 mM NaCl, 0.1% SDS, RNase inhibitor) pre-warmed to 42 °C; four 10-min elutions were performed at 42 °C with periodic vortexing. The eluates for each replicate were pooled and RNA was then purified by phenolchloroform and chloroform with EtOH precipitation (COLO320) or by column purification using New England Biolabs Monarch RNA Cleanup Kit T2030 (GBM39). Sequencing libraries were prepared using the NEBNext Small RNA Library Prep Kit (New England Biolabs, catalogue no. E7330) and sequenced by Novaseq PE150. The sequence data were mapped to human reference genome (hg38) using STAR, v.2.7.10b (ref. ^17^). HOMER (v.4.11.1) was used for de novo transcript identification on each strand separately using the default GRO-seq setting. Reads with MAPQ values less than 10 were filtered using SAMtools (v.1.8). Duplicate reads were removed using picard-tools. GRO-seq signal was converted to the bigwig format for visualization using deepTools bamCoverage^18^ (v.3.3.1) with the following parameters: --binSize 10 --normalizeUsing CPM --effectiveGenomeSize 3209286105 --exactScaling.

### Total RNA library preparation

Total RNA from each sample was isolated with Quick-RNA Miniprep Kit (Zymo Research, catalogue no. R1054) with input of 1–2 million cells. RNA libraries were constructed using TruSeq Stranded Total RNA Library Prep Kit with Ribo-Zero (Illumina, catalogue no. 20020596). Nextseq 550 sequencing system (Illumina) produced 20–30 million of ×2, 75 bp paired-end reads per sample. The sequence data were mapped to human reference genome hg38 using STAR, v.2.7.10b (ref. ^17^), following the ENCODE RNA-seq pipeline. Reads with MAPQ values less than ten were filtered using SAMtools (v.1.8). Ribo-Zero signal was converted to the bigwig format for visualization using deepTools bamCoverage^18^ (v.3.3.1) with the following parameters: --binSize 10 --normalizeUsing CPM --effectiveGenomeSize 3209286105 --exactScaling.

### KAS-seq library preparation

KAS-seq experiments were carried out as previously described^12^ with modifications^13^. Briefly, cell culture media was supplemented with 5 mM N_3_-kethoxal (final concentration), and cells were incubated for 10 min at 37 °C in a six-well plate. Genomic DNA was then extracted using the Monarch gDNA Purification Kit (NEB T3010S) following the standard protocol but with elution using 50 µl 25 mM K_3_BO_3_ at pH 7.0. Click reaction was carried out by mixing 87.5 µl purified DNA, 2.5 µl 20 mM DBCO-PEG4-biotin (dimethylsulfoxide (DMSO) solution, Sigma, catalogue no. 760749) and 10 µl 10× PBS in a final volume of 100 µl. The reaction was then incubated at 37 °C for 90 min. DNA was purified using AMPure XP beads by adding 50 µl beads per 100 µl reaction, washing beads on a magnetic stand twice with 80% EtOH and eluting in 130 µl 25 mM K_3_BO_3_. Purified DNA was then sheared using a Covaris E220 instrument down to around 200–400 bp size. Pulldown of biotin-labelled DNA was initiated by separating 10 µl of 10 mg ml^−^^1^ Dynabeads MyOne Streptavidin T1 beads (Life Technologies, catalogue no. 65602) on a magnetic stand, then washing with 180 µl of 1× Tween Washing Buffer (TWB; 5 mM Tris-HCl pH 7.5; 0.5 mM EDTA; 1 M NaCl; 0.05% Tween 20). Beads were then resuspended in 300 µl of 2× binding buffer (10 mM Tris-HCl pH 7.5, 1 mM EDTA, 2 M NaCl), sonicated DNA was added (diluted to a final volume of 300 µl if necessary) and beads were incubated for at least 15 min at room temperature on a rotator. Beads were separated on a magnetic stand and washed with 300 µl of 1× TWB and heated at 55 °C in a Thermomixer with shaking at 1,000 rpm for 2 min. The supernatant was removed on a magnetic stand and the TWB wash and 55 °C incubation were repeated.

Libraries were prepared on beads using the NEBNext Ultra II DNA Library Prep Kit (NEB, catalogue no. E7645). First, end repair was carried out by incubating beads for 30 min at 20 °C in a Thermomixer with shaking at 1,000 rpm in 50 µl 1× EB buffer plus 3 µl NEB Ultra End Repair Enzyme and 7 µl NEB Ultra End Repair Enzyme. This was followed by incubation at 65 °C for 30 min. Second, adaptors were ligated by adding 2.5 µl NEB adaptor, 1 µl ligation enhancer and 30 µl blunt ligation mix, incubating at 20 °C for 20 min, then adding 3 µl USER enzyme and incubating at 37 °C for 15 min (in a Thermomixer, with shaking at 1,000 rpm). Beads were separated on a magnetic stand and washed with 180 µl TWB for 2 min at 55 °C and 1,000 rpm in a Thermomixer. After magnetic separation, beads were washed in 100 µl 0.1× TE buffer, resuspended in 15 µl 0.1× TE buffer and heated at 98 °C for 10 min. PCR was carried out by adding 5 µl of each of the i5 and i7 NEBNext sequencing adaptors together with 25 µl 2× NEB Ultra PCR Mater Mix, with a 98 °C incubation for 30 s and 15 cycles of 98 °C for 10 s, 65 °C for 30 s and 72 °C for 1 min, followed by incubation at 72 °C for 5 min. Beads were separated on a magnetic stand and the supernatant was cleaned up using 1.8× AMPure XP beads.

Libraries were sequenced in a paired-end format on an Illumina NextSeq instrument using NextSeq 550 High-Output Kits (2 × 36 cycles). The sequence data were mapped to the hg38 assembly of the human genome using Bowtie^19,20^ with the following settings: -v 2-k 2-m 1--best--strata-X 1000. Duplicate reads were removed using picard-tools (v.1.99). MACS2 (ref. ^21^) (v.2.1.1) was used for peak-calling with the following parameters: --broad -g hs --broad-cutoff 0.01 -q 0.01. Browser tracks are generated after normalizing to input using bamCompare default setting.

### ChIP–seq library preparation

Three million cells per replicate were fixed in 1% formaldehyde for 15 min at room temperature with rotation and then quenched with 0.125 M glycine for 10 min at room temperature with rotation. Fixed cells were pelleted at 1,300*g* for 5 min at 4 °C and washed twice with cold PBS before storing at −80 °C. Membrane lysis was performed in 5 ml LB1 (50 mM HEPES pH 7.5, 140 mM NaCl, 1 mM EDTA, 10% glycerol, 0.5% IPEGAL-CA630, 0.25% Triton X-100, Roche protease inhibitors 11836170001) for 10 min at 4 °C with rotation. Nuclei were pelleted at 1,400*g* for 5 min at 4 °C and lysed in 5 ml LB2 (10 mM Tris-Cl pH 8.0, 200 mM NaCl, 1 mM EDTA, 0.5 mM EGTA, Roche protease inhibitors) for 10 min at room temperature with rotation. Chromatin was pelleted at 1,400*g* for 5 min at 4 °C and resuspended in 1 ml of TE buffer plus 0.1% SDS before sonication on a Covaris E220 with the following settings: 140 W, 10% duly, 200 cycles per burst, 600 s per sample. Samples were clarified by spinning at 16,000*g* for 10 min at 4 °C. Supernatant was transferred to a new tube and diluted with two volumes of IP dilution buffer (10 mM Tris pH 8.0, 1 mM EDTA, 200 mM NaCl, 1 mM EGTA. 0.2% Na-DOC, 1% Na-laurylsarcosine, 2% Triton X-100). Then, 50 µl of sheared chromatin was reserved as input and ChIP was performed overnight at 4 °C with rotation with 7.5 µg of H3K36me3 antibody (ab9050) (1:300 dilution). Per sample, 100 μl protein A dynabeads were washed three times with 1 ml chilled block buffer (0.5% BSA in PBS) and then added to the chromatin after overnight incubation with antibody and rotated for 4 h at 4 °C. Samples were washed five times in 1 ml pre-chilled wash buffer (50 mM HEPES pH 7.5, 500 mM LiCl, 1 mM EDTA, 1% IPEGAL-CA630, 0.7% Na-DOC) and then 1 ml pre-chilled TE + 50 mM NaCl. Samples were eluted in elution buffer (50 mM Tris pH 8.0, 10 mM EDTA, 1% SDS) at 65 °C. NaCl was added to a final concentration of 455 mM. Samples were incubated with 0.2 mg ml^−^^1^ proteinase K for 1 h at 55 °C and then decross-linked overnight at 65 °C. Samples were treated with 0.2 mg ml^−^^1^ RNAase for 2 h at 37 °C and then purified with the Zymo ChIP DNA Clean & Concentrator Kit (D2505). Libraries were prepared using the NEBNext Ultra II DNA Library Prep Kit (E7645) and sequenced by NovaSeq PE150. The sequence data were trimmed by Trimmomatic^22^ (v.0.36) to remove adaptor and then mapped to the hg38 assembly of the human genome using Bowtie2 (refs. ^19,20^) with the following settings: --local --very-sensitive --phred33 -X 1000. Reads with MAPQ values less than ten were filtered using SAMtools (v.1.8). Duplicate reads were removed using picard-tools. CHIP–seq signal was converted to the bigwig format for visualization using deepTools bamCoverage^18^ (v.3.3.1) with the following parameters: --binSize 10 --normalizeUsing CPM --effectiveGenomeSize 3209286105 --exactScaling.

### IF and DNA FISH staining

Coverslips were coated with 100 µg ml^−^^1^ poly-l-lysine overnight or 10 µg ml^−^^1^ laminin for 1 h at 37 °C before seeding cells. Asynchronized cells were seeded onto slides and subject to different treatment. Where indicated, EdU was added to each well at 10 µg ml^−^^1^ 30 min before collecting samples. IF and dual-IF DNA FISH staining were performed as described before. Briefly, slides were fixed with ice-cold 4% paraformaldehyde (PFA) for 15 min, followed by permeabilization with 0.5% Triton X-100 in PBS for 15 min at room temperature. Samples were blocked with 3% BSA in PBS for 1 h at room temperature before incubation with primary antibody diluted in blocking buffer at 4 °C overnight. Dilution ratio for first antibodies was as follows: γH2Ax, 1:500; pRPA2-S33, 1:1,000; pCHK1S345, 1:250; 53BP1, 1:500; cyclin A, 1:100; pRNAPII S2/S4, 1:1,000. After washing with PBS a total of three times for 5 min each, slides were incubated with secondary antibody diluted in blocking buffer at room temperature for 1 h. Samples were fixed with ice-cold 4% PFA for 20 min after washing with PBS. If combined with DNA FISH staining, fixed samples were further permeabilized with ice-cold 0.7% Triton X-100 per 0.1 M HCl (diluted in PBS) for 10 min on ice. DNA was denatured by 1.5 M HCl for 30 min at room temperature, followed by dehydration in ascending ethanol concentration. Diluted FISH probes (Empire Genomics) were pre-denatured at 75 °C for 3 min and added onto air-dried slides. After incubation at 37 °C overnight, slides were washed with 2× SSC to get rid of non-specific binding, followed by DAPI staining. Where indicated, EdU staining was performed with the Click-iT Plua EdU Alexa Fluor 647 Imaging Kit (Invitrogen, catalogue no. C10640).

### Validation of PC3-DM and PC3-HSR cell lines

Genomic DNA was extracted from a confluent six-well dish using the QIAamp DNA Mini Kit (Qiagen) according to the manufacturer’s protocol. Briefly, single cells were collected and resuspended in 200 µl 1× PBS, followed by the addition of 20 µl QIAGEN proteinase K and 200 µl buffer AL. The mixture was pulse-vortexed for 15 s and incubated at 56 °C for 10 min. A volume of 200 µl absolute ethanol was added to the sample and pulse-vortexed for 15 s. The entire mixture was pipetted into a QIAamp Mini spin column and centrifuged at 6,000*g* for 1 min. Filtrate was discarded and 500 µl buffer AW1 was added to the column. After centrifugation at 6,000*g* for 1 min, the column was subjected to another round of wash with 500 µl buffer AW2. The filtrate was discarded after centrifugation at full speed for 3 min. The column was then placed in a clean 1.5 ml microfuge tube and 50 µl of buffer AE was added to reconstitute genomic DNA after centrifugation at 6,000*g* for 1 min.

WGS library preparation was performed with the FS DNA Library Prep Kit from NEB according to the manufacturer’s protocol, with these parameters in place: (1) 250 ng gDNA was used as input; (2) fragmentation was done with an incubation time of 18 min to yield 200–450 bp fragments; (3) the final library size distribution was between 320–470 bp (that is, first bead selection was done with a bead volume of 30 µl and second bead selection was done with a bead volume of 15 µl); (4) the final PCR amplification was performed for four cycles. PE150 sequencing was performed on NovaSeq to yield at least 10× coverage at Novogene. Adaptor sequences were removed from raw fastq files using Trim Galore at default settings, followed by alignment to the hg38 reference genome using Map with BWA-MEM to generate the BAM files. BAM files were then uploaded to the GenePattern Notebook for AmpliconArchitect analysis under default settings.

### ecDNA structure analysis

We utilized the AmpliconSuite-pipeline (v.1.2.2, https://github.com/AmpliconSuite/AmpliconSuite-pipeline), which invoked CNVKit (v.0.9.9)^43^, AmpliconArchitect^44^ (AA; v.1.3.r8) and AmpliconClassifier^3^ (AC; v.1.1.2). In brief, the analysis pipeline first identifies seed regions of focal amplification from whole-genome copy number calls, then among the seed regions AA analyses copy number and structural variation jointly to construct a local genome graph encoding structural rearrangements and copy numbers. AA then extracts genome paths and cycles from the genome graph that explain the observed changes in copy number and structural variation. The outputs of AA are passed to AC, which applies a rule-based method to match the patterns of copy number, structural variation and structures extracted from the genome graph to known types of focal amplifications, such as ecDNA. To minimize sequencing artefacts derived from insert size distribution variance, we set the AmpliconSuite-pipeline argument --AA_insert_sdevs 9. For PC3 samples, --downsample 1 was also set to reduce additional sequencing artefacts. Default parameters were used otherwise.

For COLO320DM/HSR, we utilized the general ecDNA regions and the candidate ecDNA structure from ref. ^10^, after lifting over coordinates to hg38. For GBM39ec/HSR and PC3-DM/HSR, ecDNA regions were derived from AA output files. From the DM samples, regions with copy number greater than ten in the AA amplicon containing the ecDNA of interest were defined as the ecDNA regions. In GBM39ec/HSR, we also included the vIII deletion in the ecDNA region. Candidate ecDNA structures were derived from the AA cycle with highest assigned copy count containing the oncogene of interest (GBM39: *EGFR* and PC3: *MYC*). For GBM39, the ecDNA structure was consistent with a previously published reconstruction^11^. Circular ecDNA visualizations were generated with CycleViz (https://github.com/AmpliconSuite/CycleViz). Gene and focal amplification copy numbers were derived from the AA graph file and the AC feature basic properties file, respectively. Structural similarity scores of the focal amplifications were computed using the feature_similarity.py script in AC, which computes a similarity score based on the overlapping genomic boundaries and shared structural variants between two focal amplifications. For the PC3 samples, we utilized the related amplicon_similarity.py script to obtain similarity scores, as the exact boundaries of the ecDNA could not be easily resolved with AC.

### Replication combing assay

Replication fork speed in ecDNA was evaluated using the molecular combing assay. COLO320DM and COLO320HSR cells were seeded into plates and allowed to grow into log phase, nascent DNA synthesize was pulse labelled with thymidine analogues: CldU and IdU sequentially for equal amount of time. Following pulse labelling, cells were harvested and embedded into agarose plugs using the Genomic Vision FiberPrep Kit (Genomic Vision). DNA extraction, combing and immunostaining was performed according to the EasyComb service procedures (Genomic Vision). Coverslips were scanned with a FiberVision scanner and images were analysed using FiberStudio software (Genomic Vision). Fork speed was calculated using replication signals with contiguous CldU–IdU tracks. Only intact signals, flanked by counterstaining of the DNA fibre, were selected for analysis.

### Locus-replication combing assay

DNA replication activity at the *MYC* loci was assessed using molecular combing assay. COLO320DM and COLO320HSR cells were seeded into plates and allowed to grow into log phase, nascent DNA synthesize was pulse labelled with thymidine analogues: CldU and IdU for equal amount of time. Following pulse labelling, cells were harvested and embedded into agarose plugs using the Genomic Vision FiberPrep kit (Genomic Vision). DNA extraction and combing was performed according to the EasyComb service procedures (Genomic Vision). DNA-labelled FiberProbes (Genomic Vision) targeting *MYC* loci were produced and hybridized to combed DNA. Correspondence between theoretical and experimental probe coverage patterns was validated by measuring hybridized probe length in control samples. After immunostaining of replication signals and DNA probes, coverslips were scanned with a FiberVision scanner. Image analysis and measurements were performed using FiberStudio software (Genomic Vision). Fork speed was calculated using replication signals with contiguous CldU–IdU tracks.

### Comet-FISH

Alkaline comet-FISH assays were performed according to the literature, with minor modifications^45,46^. Cells were harvested by trypsinization, washed with PBS and placed on ice. Cells were diluted in 37 °C low melting point (LMP) agarose (IBI Scientific) in PBS to a final concentration of 0.7% and spread on precoated glass slides with a coverslip. Overnight lysis was performed at 4 °C in alkaline lysis solution (2.5 M NaCl, 100 mM EDTA, 10 mM Tris pH 10, 1% Triton X-100, 10% DMSO) protected from light. The following day, slides were equilibrated for 30 min in alkaline electrophoresis buffer (200 mM NaOH, 1 mM EDTA, pH less than 13) in a Coplin jar and subsequently electrophoresed at 25 V for 30 min. Slides were then neutralized with Tris, dehydrated in 70% ethanol and dried at room temperature.

To detect ecDNA through FISH, Cy5-labelled probes were generated from RP11-440N18 BAC DNA sonicated to 150 bp and labelled using a DNA labelling kit (Mirus Bio). Slides were denatured with 0.5 M NaOH for 30 min at room temperature, dehydrated in an ethanol series (70%, 85%, 95%) and allowed to dry at room temperature. The hybridization mixture containing probe DNA (200 ng per slide) and Cot-1 DNA (8 μg per slide) was denatured separately at 75 °C for 10 min and pre-annealed at 37 °C for 1 h. Probe was added to the slides and spread with a glass coverslip and incubated at 37 °C overnight in a humidified chamber. The following day, slides were washed four times in 2× SSC, 50% formamide at 42 °C and subsequently washed twice in 2× SSC at 42 °C. Slides were dipped briefly in 70% ethanol and air-dried. Slides were mounted with Everbrite (Biotium) containing SYBR Gold (Invitrogen) diluted 1:10,000 and sealed with nail polish. Images were collected on a Nikon Eclipse TE2000-E using a ×60 oil objective.

### Cell viability assay

Cell viability assay was performed using CellTiter-Glo (Promega, catalgoue no. G8461) as previously described^47^. Briefly, cells were seeded into a 384-well plate one day before adding inhibitors. Equal volumes of vehicles or drugs diluted at indicated concentration were added into each well the next day, and the cells were incubated for three days. On the third day, after equilibrating plate and CellTiter-Glo reagent at room temperature for 30 min, reagent was added into each well and incubated for 15 min at room temperature. Luminescence was measured using a Synergy 2 microplate reader. Four biological replicates were performed for each condition. Data analysis was performed with GraphPad Prism (v.9.1.0).

### TUNEL

TUNEL assay (Invitrogen, catalogue no. C10617) was performed to detect DNA fragmentation during apoptosis. COLO320DM, COLO320HSR and SNU16 cells were treated with 1 µM CHIR-124 for indicated times. All cells including floating cells were collected and spun down onto slides using a cytospin (Thermo Scientific Cytospin 4 Centrifuge). Slides were fixed with 4% PFA and permeabilized with 0.25% Triton X-100, followed by labelling of free double strand end with EdUTP by reaction catalysed by TdT enzyme in a humidified chamber at 37 °C for 60 min. Incorporated EdUTP was detected through Click-iT Plus TUNEL reaction according to the manufacturer’s manual at 37 °C for 30 min. Slides were counterstained with DAPI and mounted with ProLong Diamond Antifade.

### Annexin V staining

Cell apoptosis was detected through flow cytometry using a FITC Annexin V Apoptosis Detection Kit (BD Biosciences, catalogue no. 556547). Cells were treated with 1 µM of CHIR-124 for the indicated time, and all the cells including floating cells were collected. After washing with PBS twice and cell number counting, cells were resuspended in 1× binding buffer provided by the kit at a concentration of 1 × 10^6 ^cells per millilitre. One hundred microlitres of the cell suspension was transferred to a FACS tube and stained with FITC Annexin V and propidium iodide. After incubation at room temperature for 15 min, all the samples were analysed with BD LSR II flow cytometry (BD Biosciences) within 1 h. Flow cytometry data were analysed through Beckman Coulter Kaluza software (v.2.1).

### Microscope and image analysis

Images were taken by conventional fluorescence microscopy or confocal microscopy. Conventional fluorescence microscopy was performed on a Leica DMi8 widefield microscope by Las X software (v.3.8.2.27713) using a ×63 oil objective. Confocal microscopy was performed on a ZEISS LSM 880 inverted confocal microscope using ZEN (black v.2.3) (Stanford CSIF Facility). Z-stacks were taken for each field of view and a best-in-focus stack was identified for downstream image analysis, except for Fig. 3a, where a max projection was performed by ImageJ (v.1.53t).

Image analysis and quantification were performed using the open-source software CellProfiler (v.4.2.1). For foci number analysis, DAPI staining, IF staining and DNA FISH channel were analysed through automatic thresholding and segmentation to cell nuclei, pRPA2S33/γH2AX foci and DNA FISH foci respectively. Colocalization was performed using an object-based colocalization method. For fluorescence intensity measurement, nuclei were called based on DAPI channel through automatic thresholding and segmentation; mean fluorescence intensity was retrieved by measuring mean fluorescence intensity within each nucleus.

### RS score computation

#### RS score 1

The gene set variation analysis^48^ was utilized to assess the enrichment of the DNA RS response (RSR) gene set^20^ in TCGA samples using RNA-seq data^49^. The RSR gene set was curated based on genes affected by defects in the DNA RS response. RNA-seq transcripts per kilobase million values for TCGA samples were retrieved from the GDC data portal^49^. Gene set variation analysis generated enrichment scores for both up- and down-regulated RSR genes. The final RSR score was determined as the difference between the up and down enrichment scores.

#### RS score 2

The RS signature score of each sample from TCGA was retrieved from the literature from ref. ^21^, which was transformed linearly between zero and one by subtracting the minimum score and dividing by the maximum score. TCGA sample ecDNA status classification was performed as stated in a previous publication^1^.

#### Both methods

Briefly, 1,921 TCGA samples were grouped into five subtypes by AC (https://github.com/AmpliconSuite): ecDNA, breakage–fusion–bridge, complex non-cyclic, linear and no amplification. Samples with a break–fusion–bridge or complex non-cyclic status were removed from the analysis due to the challenges of detecting ecDNA from short-read data. Samples with linear amplification and no amplification were classified as ecDNA^−^. After removing metastasis sample and ecDNA^−^ samples without matching ecDNA^+^ samples of the same tissue origin, a total of 232 ecDNA^+^ and 582 ecDNA^−^ samples were included in the analysis.

### CRISPR experiment

sgRNA template oligos targeting the gene encoding CHK1 was synthesized (Integrated DNA Technologies) and was ligated into a CRISPR expression vector with red fluorescent protein (RFP) (Cellecta-pRSG16-U6-sg-HTS6C-UbiC-TagRFP-2A-Puro). Non-targeting green fluorescent protein (GFP) (sgNT-GFP) plasmid was purchased.

ecDNA^+^ and ecDNA^−^ Hela cells were transduced with sgCHK1-RFP or sgNT-GFP virus, and puromycin (Sigma) was added at 2.5 µg ml^−^^1^ for selection for 48 h. After 48 h of puromycin selection (day 0), an equal number of cells expressing either sgCHK1-RFP or sgNT-GFP were mixed to obtain the RFP to GFP population ratio. In the following days, flow cytometry analysis was performed to determine the sgCHK1-RFP to sgNT-GFP ratio. The mixed cell population cultures were maintained at subconfluency. The sgRNA sequences targeting CHK1 were as follows:

No. 17: CCTGACAGCTGTCACTGGGT

No. 18: GCTGTCAGGAGTATTCTGAC

### Western blotting

Samples were lysed in radioimmunoprecipitation assay buffer (Boston BioProducts, catalogue no. BP-115) supplemented with protease/phosphatase inhibitors (Fisher Scientific, catalogue no. 78444). Protein concentration was quantified with bicinchoninic acid assay (Fisher Scientific, catalogue no. 23225) and samples were prepared in 4× sample buffer (Bio-Rad, catalogue no. 1610747). Samples were loaded and run on 4–12% Bis-Tris Gradient Gel (Fisher Scientific, catalogue no. WG1403BOX) and transferred onto a nitrocellulose membrane (Bio-Rad, catalogue no. 1704271). The membrane was blocked with 5% BSA in Tris-buffered saline with Tween (Fisher Scientific, catalogue no. 28360) for an hour, and then primary antibody (1:1,000 dilution) was added and incubated overnight at 4 °C. Following primary antibody incubation, the membrane was washed with Tris-buffered saline with Tween and incubated with secondary antibody for 1 h. The membrane was then incubated with enhanced chemiluminescence reagent (Fisher Scientific, catalogue no. 32106) and image acquisition was performed on ProteinSimple FluorChemE.

### Detection of phosphorylated CHK1 Ser345 using the AlphaLisa SureFire assay

Compound activity in cells was measured using an AlphaLISA SureFire Ultra p-CHK1 (Ser345) assay (Perkin Elmer, catalogue no. ALSU-PCHK1-A10K). HT29 cells were cultured in McCoy 5 A medium with 10% FBS and 1% penicillin-streptomycin and seeded to 96-well plates (Corning, catalogue no. 3599). Compounds were serially diluted in DMSO over a 10-point dose range with 3-fold dilution, and compound solution was added to each well containing cells. Plates were centrifuged at 1,000 rpm for 30 s. Plates were incubated at 37 °C for 16 h. Supernatant was removed by flicking the plate against a paper towel. Wells were washed once with PBS solution. To each well was added freshly prepared lysis buffer and plates were agitated on a plate shaker at 400 rpm for 30 min. The 96-well cell plates were centrifuged at 1,500 rpm for 1 min. From each well was transferred 10 µl of the lysates to a 384-well Optiplate (Perkin Elmer, catalogue no. 6007290). To each well was added Acceptor Mix (5 µl) and the plates were sealed and wrapped in foil. Plates were agitated on a plate shaker for 2 min, then incubated at room temperature for 1 h. To each well was added Donor Mix (5 µl) and the plates were sealed and wrapped in foil. Plates were agitated on a plate shaker for 2 min, then incubated at room temperature for 1 h. AlphaLisa signal was read on an EnVision multimode plate reader (Perkin Elmer). Data were fitted to dose–response curves using XLfit (IDBS) or GraphPad Prism (GraphPad software) to calculate IC_50_ values for each compound tested.

### Kinase HTRF biochemical assay

CHK1 enzyme activity was measured using a homogeneous time resolved fluorescence (HTRF) KinEASE assay (Cisbio, catalogue no. 62ST1PEC). Full-length human CHK1 protein (GenBank accession number NP_001265.1) was obtained from Carna Biosciences, Inc. (catalogue no. 02-117). The enzyme reaction was carried out in assay buffer containing (final concentrations): CHK1 enzyme (0.012 ng µl^−^^1^), MgCl2 (5 mM) and DTT (1 mM). To determine compound dose response, DMSO stock solutions were serially diluted in a ten-point concentration series in duplicate. Compound solution (50 nl) was added to 384-well assay plates (Greiner, catalogue no. 784075). To each well containing compound solution was added assay buffer solution (5 µl). Plates were centrifuged at 1,000 rpm for 1 min, then incubated at room temperature for 10 min. The reaction was started by addition of substrate buffer (5 µl per well) containing (final concentrations): STK substrate 1-biotin (120 nM) and ATP (1 mM). Assay plates were centrifuged at 1,000 rpm for 1 min, then incubated at room temperature for 60 min. The reaction was stopped by the addition of detection buffer (Cisbio, 10 µl) containing (final concentrations): STK antibody cryptate (0.25 nM) and streptavidin-XL665 (7.5 nM). Plates were centrifuged at 1,000 rpm for 1 min, then incubated at 25 °C for 2 h. HTRF signal was read on an EnVision multimode plate reader (Cisbio) in HTRF mode. Data were fit to dose–response curves using XLfit (IDBS) or Prism (GraphPad Software) to calculate IC_50_ values for each compound tested.

### Phospho-RPA32 S8 IF high content imaging

Optical-bottom 96-well plates (Thermo Scientific, catalogue no. 165305) were coated with 50 µl of 1:1 poly-l-lysine (R&D Systems, catalogue no. 3438-100-01) and poly-d-lysine (R&D Systems, catalogue no. 3439-100-01) for 3 h at room temperature. The wells were washed once with 100 µl of PBS (Gibco, catalogue no. 10010-023) and all liquid was removed from the wells and allowed to dry fully at room temperature. COLO320 ecDNA^+^ cells were seeded at 15,000 cells per well in 100 µl of Roswell Park Memorial Institute media (Thermo Fisher, catalogue no. 22400089) supplemented with 10% FBS (Omega Scientific, catalogue no. FB-01). Cells were left to attach in a 37 °C incubator with 5% CO_2_ overnight. The following day, cells were treated with BBI-825 for 16 h. Following treatment, all culture media was removed, and cells were fixed with 4% PFA (Boston BioProducts, catalogue no. BM-155) for 15 min at room temperature. After fixation, the 4% PFA was removed and wells were washed twice with 100 µl of PBS. The cells were then permeabilized with 100 µl of 0.5% Triton X-100 (Sigma-Aldrich, catalogue no. T8787) in PBS for 15 min at room temperature. After permeabilization, wells were washed twice with 100 µl of PBS and then blocked with 5% goat serum (Abcam, catalogue no. ab7481) and 1 mg ml^−^^1^ of BSA (GeminiBio, catalogue no. 700-100 P) for 1 h at room temperature. The primary antibody (phospho-RPA32 (S8); Cell Signaling, catalogue no. 54762) was diluted at 1:200 in blocking buffer and 50 µl was added to all wells and incubated at 4 °C overnight. Plates were then washed three times with 100 µl of PBS and then incubated with 1:1,000 dilution of secondary antibody (Goat anti-Rabbit IgG Alexa Fluor Plus 594; Thermo Fisher, catalogue no. A32740s) and 1:1,000 dilution of Hoechst 33342 (Biotium, catalogue no. 40046) in blocking buffer for 1 h at room temperature. Plates were then washed three times with 100 µl of PBS; 100 µl of PBS was left in the wells following the final wash. The plate was imaged using a CellInsight CX7 LZR Pro High Content imager (Thermo Fisher Scientific) and data analysed using the Spot Detector BioApplication module on the HCS Studio Cell Analysis software (Thermo Fisher Scientific). Puncta were detected using a pixel thresholding method within a nucleus, and cells that contained three or more puncta of phosphorylated RPA32 Ser8 staining were considered as a positive signal.

### Xenograft

Animal experiments were performed in accordance with protocols approved by the Charles River Accelerator and Development Lab (CRADL) Institutional Animal Care and Use Committee (protocol no. EB17-010-066). Mice were socially housed in individually ventilated cages on a 12/12 h light/dark cycle with temperatures between 65 and 75 °F and 30–50% humidity. The SNU16 gastric cancer cell line was purchased from ATCC (catalogue no. CRL5974) and maintained in Roswell Park Memorial Institute growth medium (Gibco, catalogue no. 22400-089) supplemented with 10% FBS (Omega Scientific, catalogue no. FB-02). To establish tumours, 1 × 10^6 ^SNU16 cells in 200 µl of a 1:1 mixture of PBS and Matrigel (Corning, catalogue no. 354234) were given by subcutaneous injection into the right flank of 9-week-old female severe combined immunodeficient beige mice (Envigo, strain code 186). Tumour measurements were taken two to three times per week and body weights were taken daily. Tumour volume measurements were obtained using digital calipers and tumour volumes (mm^3^) were determined using the formula: tumour volume = (*L* × *W*2)/2, where *L* is the length/largest tumour diameter and *W* is the width/shortest tumour diameter, with all tumours collected before reaching 1,500 mm^3^. Animals (eight mice per group, which historically allowed for significance determination between vehicle and infigratinib) were randomly assigned to unblinded treatment with vehicle, infigratinib (15 mg kg^−^^1^ oral (PO) once-daily (QD)), BBI-2779 (30 mg kg^−^^1^ PO every other day (Q2D)) or the combination of BBI-2779 and infigratinib once average tumour volume was 285 (±10)/mean (±s.e.m.) mm^3^. One vehicle tumour was taken down on day 22; the mouse was sacrificed due to large tumour volume. Infigratinib was formulated in a 1:1 mixture of sodium acetate buffer, pH 4.6 and polyethylene glycol 300. BBI-2779 was formulated in 0.5% methylcellulose (Sigma-Aldrich, catalogue no. M0512) and 0.2% Tween 80 (AG Scientific, catalogue no. T-2835) in HyPure Molecular Biology Grade Water (HyClone, catalogue no. SH30538.02). Dose holidays were provided to individual animals that demonstrated greater than −10% body-weight change from baseline, and Nutra-Gel was provided to the entire treatment group. Animals were sacrificed 6 h, 24 h or 36 h after the last dose, and tumours were collected for western blot or copy number analysis.

### Copy number analysis from xenograft samples

For copy number analysis, tumours were cut into 10–20 mg pieces and flash-frozen in liquid nitrogen. DNA was extracted using the QIAcube DNA Extraction Kit (Qiagen, no. 51331). Briefly, a mixture of buffer ATL and proteinase K was added to the frozen tumour pieces, and they were set out to equilibrate to room temperature. Tumours were then vortexed for 30 s and placed into an incubator at 56 °C to digest overnight. The next morning, an additional 150 μl of buffer ATL was added and samples vortexed for an additional 30 s to reduce the viscosity of the samples before transfer to the S block. Qiagen protocol for the 96 QIAcube HT was followed for the remainder of the DNA isolation. Purified DNA was quantified for the presence of double-stranded DNA on the QIAxpert (Qiagen, catalogue no. 9002340). The DNA was diluted to 5 ng µl^−^^1^ (5× working stock) in RNase/DNase free water (Thermo Fisher Scientific, catalogue no. 10977015) and 2 µl was loaded into a 384-well plate. Master mix recipe (Master Mix (2×), 5.5 µl; CNA (Target Gene) 20×, 0.55 µl; CNR telomerase reverse transcriptase (TERT) 20×, 0.55 µl; nuclease-free water, 2.2 µl) was made containing TaqPath Pro Master Mix 2× (Thermo Fisher Scientific, catalogue no. A30866) human female genomic DNA (Promega, catalogue no. G1521) as a reference, internal controls (human TERT) and FGFR2 or *MYC* target gene probe (Thermo Fisher Scientific, catalogue no. 4400292). Reactions were run on the QuantStudio 6 or 7 (Thermo Fisher Scientific) using the qPCR reaction settings as follows: denature/enzyme activation: 95 °C, 10 min; 40 cycles of denature 95 °C, 15 s; anneal/extend 60 °C, 60 s.

### Quantifications and statistical analysis

All statistical methods and sample size have been stated in figure legends or the Methods section. No statistical methods were used to predetermine the sample size. The default test type was a two-sided statistic test, unless indicated in the text. The investigators were not blinded to allocation during experiments and outcome assessment.

### Reporting summary

Further information on research design is available in the Nature Portfolio Reporting Summary linked to this article.

## Online content

Any methods, additional references, Nature Portfolio reporting summaries, source data, extended data, supplementary information, acknowledgements, peer review information; details of author contributions and competing interests; and statements of data and code availability are available at 10.1038/s41586-024-07802-5.

## Supplementary information

Supplementary Information The Supplementary Information file contains the Supplementary Methods, which describe the details of the step-by-step synthesis of compound BBI-2779.

Reporting Summary

## Source data

Source Data Fig. 4 and Source Data Extended Data Figs. 9 and 10

## Data Availability

GRO-seq, Ribo-Zero RNA-seq, ChIP–seq, WGS and KAS-seq data generated in this study can be accessed by GEO under accession number https://www.ncbi.nlm.nih.gov/geo/query/acc.cgi?accGSE249657. AA outputs and copy numbers of COLO320, GBM39 and PC3 can be accessed at https://ampliconrepository.org/project/6639560c48cbf4a5ccffad4d. WGS data of COLO320 and GBM39 used in this study are publicly available under SRA: https://www.ncbi.nlm.nih.gov/bioproject/?term=PRJNA506071^11^). BBI-2779 is available upon request to C.A.H. at Boundless Bio. Source data are provided with this paper.
