# Supplementary material for: Enhancing transcription–replication conflict targets ecDNA-positive cancers
Source: Nature. 2024 Nov 6;635(8037):210–8. doi: 10.1038/s41586-024-07802-5 (PMC11540844; doi:10.1038/s41586-024-07802-5)
Supplement: Supplementary file 1 — The Supplementary Information file contains the Supplementary Methods, which describe the details of the step-by-step synthesis of compound BBI-2779. [file 41586_2024_7802_MOESM1_ESM.docx]

**Supplementary Information**

**Synthesis of BBI-2779**

All chemicals were purchased from commercial suppliers and used as received unless otherwise indicated. Proton nuclear magnetic resonance (1H NMR) spectra were recorded on Bruker AVANCE 400 MHz spectrometers. Chemical shifts are expressed in d ppm and are calibrated to the residual solvent peak: proton (CDCl_3_, 7.26 ppm). Coupling constants (J), when given, are reported in hertz. Multiplicities are reported using the following abbreviations: s = singlet, d = doublet, dd = doublet of doublets, t = triplet, q = quartet, m = multiplet (range of multiplet is given), br = broad signal, and dt = doublet of triplets. Carbon nuclear magnetic resonance (13C NMR) spectra were recorded using a Bruker AVANCE HD spectrometer at 100 MHz. Chemical shifts are reported in parts per million (ppm) and are calibrated to the solvent peak: carbon (CDCl3, 77.23 ppm).

All final compounds were purified by reverse phase HPLC or SFC. The purity for test compounds was determined by HPLC on a SHIMADZU LC-2010A HT instrument. HPLC conditions were as follows: XBRIDGE C18 3.5um 2.1*50mm,  H2O(0.05%TFA)-ACN(0.05%TFA) , ACN from 0 to 60% over 7 minutes , 7-8min, ACN from 60% to 100%, , flow rate 0.8 mL/min, UV detection (λ =214, 254 nm). The mass spectra were obtained using LCMS on a LCMS-Agilent 6125 instrument using electrospray ionization (ESI). LCMS conditions were as follows: Column: Waters Cortecs C18+, 2.7um 30 mm; Mobile phase : ACN (0.05% FA)-Water (0.05% FA); Gradient: 5% ACN to 95% ACN in 1.0 min, hold 1.0 min, total 2.5 min; Flow 1.8 mL / min; UV detection (λ = 214, 254 nm). Column Temp:45 degree. The SFC purity for test compounds was determined with a SFC Thar prep 80

Step 1: 2-bromocyclobutan-1-one (**2**)

To a solution of cyclobutanone (10 g, 0.142 mol) in CHCl_3_ (100 mL) was added Br_2_ (22.82 g, 0.170 mmol) at 0 ^o^C. The reaction mixture was stirred at 25 ^o^C for 12 hours. The residue was quenched by saturated aqueous sodium thiosulfate (100 mL) and extracted with ethyl acetate (100 mL x 3). The organic layers were combined and dried over Na_2_SO_4_ and concentrated to give 2-bromocyclobutan-1-one (19.5 g, 91.9% yield) as a colorless oil, which was used in the next step without further purification.

Step 2: 2-(2-bromo-3-methoxyphenoxy)cyclobutan-1-one (**3**)

A solution of 2-bromo-3-methoxyphenol (3 g, 0.016mol) , 2-bromocyclobutan-1-one (7.068 g, 0.080 mol) and K_2_CO_3_ (5.447 g, 0.040mol) in DMF(30 mL) was stirred at 50 ^o^C for 12 hours. The mixture was filtered and concentrated under reduced pressure. After concentration, the residue was purified via flash column chromatography, eluted with petroleum ether/ethyl acetate (from 0% to 40%) to give 2-(2-bromo-3-methoxyphenoxy)cyclobutan-1-one (1.65 g, 42.3 % yield) as a white solid. MS (ESI): mass calcd. for C_11_H_11_BrO_3_ 269.99, 271.99 m/z found 270.9，272.9 [M+H]^+^. LCMS (method 1, 2.5 min formic acid): Rt = 1.303 min

Step 3: N-((1R,2R)-2-(2-bromo-3-methoxyphenoxy)cyclobutyl)-2-methylpropane-2-sulfinamide (**4**)

To a solution of 2-(2-bromo-3-fluorophenoxy)cyclobutan-1-one (200 mg, 0.7720 mmol), (S)-2-methylpropane-2-sulfinamide (102.92 mg, 0.8492 mmol) and Ti(OEt)_4_ (329.13 mg, 1.158 mmol) in THF was stirred under nitrogen for 2 hours then NaBH_4_ ( 58.41 mg, 1.544 mmol ) was added at 0 ^o^C. The reaction mixture was stirred at 50 ^o^C for 2 hours. The reaction was quenched by MeOH (5 mL) and H_2_O (50 mL), then extracted with ethyl acetate (50 mL x 3). The organic layers were combined and dried over Na_2_SO_4,_ and concentrated under reduced pressure at 30 ^o^C. The residue was purified by Prep-HPLC (Daisogel-C18-10-100, 30 x 250 mm, 5 um, mobile phase: ACN--H2O (0.1%FA), gradient: 5 ~ 95) to afford 1: N-((1R,2R)-2-(2-bromo-3-methoxyphenoxy)cyclobutyl)-2-methylpropane-2-sulfinamide (30 mg, 12.1 % yield) as a white solid. MS (ESI): mass calcd. for C_15_H_22_BrNO_3_S 375.05, 377.05, m/z found 376.0, 378.0[M+H]^+^. LCMS (method 1, 2.5 min formic acid): Rt = 1.320 min.

Step 4: tert-butyl3-((tert-butoxycarbonyl)(5-cyanopyrazin-2-yl)amino)-5-(2-((1R,2R)-2-((tert-butylsulfinyl)amino)cyclobutoxy)-6-methoxyphenyl)-1H-pyrazole-1-carboxylate (**5**)

A solution of N-((1R,2R)-2-(2-bromo-3-methoxyphenoxy)cyclobutyl)-2-methylpropane-2-sulfinamide (200 mg, 0.5490 mmol), tert-butyl-3-((tert-butoxycarbonyl)(5-cyanopyrazin-2-yl)amino)-5-(4,4,5,5-tetramethyl-1,3,2-dioxaborolan-2-yl)-1H-pyrazole-1-carboxylate ( 338.87 mg, 0.6588 mmol) and K_3_PO_4_ (349.61 mg,1.6470 mmol) in dioxane : H_2_O = 5 : 1, (11 mL) was stirred under nitrogen at 100 ^o^C. Then, X-phos (104.69 mg, 0.2196 mmol) and Pd_2_(dba)_3_ (100.55 mg, 0.1098 mmol) were added. The reaction mixture was stirred at 100 ^o^C for 3 hours. The mixture was filtered and concentrated under reduced pressure. After concentration, the residue was purified via flash column chromatography, eluted with DCM/MeOH (from 0% to 10%) to give tert-butyl3-((tert-butoxycarbonyl)(5-cyanopyrazin-2-yl)amino)-5-(2-((1R,2R)-2-((tert-butylsulfinyl)amino)cyclobutoxy)-6-methoxyphenyl)-1H-pyrazole-1-carboxylate (150 mg, 40.7 % yield) as a orange solid. MS (ESI): mass calcd. for C_33_H_43_N_7_O_7_S 681.29, m/z found 682.2 [M+1]^+^. LCMS (method 1, 2.5 min formic acid): Rt = 1.402min

Step 5: 5-((5-(2-((1R,2R)-2-aminocyclobutoxy)-6-fluorophenyl)-1H-pyrazol-3-yl)amino)pyrazine-2-carbonitrile (**BBI-2779)**

To a solution of tert-butyl3-((tert-butoxycarbonyl)(5-cyanopyrazin-2-yl)amino)-5-(2-((1R,2R)-2-((tert-butylsulfinyl)amino)cyclobutoxy)-6-fluorophenyl)-1H-pyrazole-1-carboxylate (200 mg, 0.3505 mmol) in HCl in dioxane (4M, 10 mL) was stirred at 25 ^o^C for 1 hour. It was the concentrated under reduced pressure. The residue was purified by Prep-HPLC (Daisogel-C18-10-100, 30 x 250 mm, 5 um, mobile phase: ACN--H2O (0.1%FA), gradient: 5 ~ 95) to give 5-((5-(2-((1R,2R)-2-aminocyclobutoxy)-6-methoxyphenyl)-1H-pyrazol-3-yl)amino)pyrazine-2-carbonitrile (25 mg, 19.5% yield) as a white solid. MS (ESI): mass calcd. for C_19_H_19_N_7_O_2_ 377.16, m/z found 378.0 [M+H]^+^. LCMS (method 1, 2.5 min formic acid): Rt = 1.005 min ^1^H NMR (400 MHz, DMSO-*d*_6_) d ppm 10.72 (s, 1H), 8.65 (d, *J* =0.8 Hz, 1H), 8.57 (s, 1H), 8.33 (s, 1H), 7.30 (1 H, t, *J* = 8.4 Hz, 1H), 6.93 (s, 1H), 6.76 (t, *J =* 8.8 Hz, 1H), 4.66-4.46 (m, 1H), 3.82 (s, 3H), 3.59-3.56 (m, 1H), 2.35-2.30 (m, 1H), 2.12-2.05 (m, 1 H), 1.62-1.49 ( m, 2H).
