# Supplementary material for: Enhancing transcription–replication conflict targets ecDNA-positive cancers
Source: Nature. 2024 Nov 6;635(8037):210–8. doi: 10.1038/s41586-024-07802-5 (PMC11540844; doi:10.1038/s41586-024-07802-5)
Supplement: Supplementary file 2 — Reporting Summary [file 41586_2024_7802_MOESM2_ESM.pdf]

nature portfolio

Corresponding author(s): Christian A. Hassig, Paul S. Mischel, Howard Y. Chang

Last updated by author(s): May 20, 2024

Reporting Summary

Nature Portfolio wishes to improve the reproducibility of the work that we publish. This form provides structure for consistency and transparency in reporting. For further information on Nature Portfolio policies, see our Editorial Policies and the Editorial Policy Checklist.

Statistics

For all statistical analyses, confirm that the following items are present in the figure legend, table legend, main text, or Methods section.

n/a	Confirmed	
☐	☒	The exact sample size (*n*) for each experimental group/condition, given as a discrete number and unit of measurement
☐	☒	A statement on whether measurements were taken from distinct samples or whether the same sample was measured repeatedly
☐	☒	The statistical test(s) used AND whether they are one- or two-sided<br>*Only common tests should be described solely by name; describe more complex techniques in the Methods section.*
☒	☐	A description of all covariates tested
☐	☒	A description of any assumptions or corrections, such as tests of normality and adjustment for multiple comparisons
☐	☒	A full description of the statistical parameters including central tendency (e.g. means) or other basic estimates (e.g. regression coefficient) AND variation (e.g. standard deviation) or associated estimates of uncertainty (e.g. confidence intervals)
☐	☒	For null hypothesis testing, the test statistic (e.g. *F*, *t*, *r*) with confidence intervals, effect sizes, degrees of freedom and *P* value noted<br>*Give P values as exact values whenever suitable.*
☒	☐	For Bayesian analysis, information on the choice of priors and Markov chain Monte Carlo settings
☐	☒	For hierarchical and complex designs, identification of the appropriate level for tests and full reporting of outcomes
☒	☐	Estimates of effect sizes (e.g. Cohen's *d*, Pearson's *r*), indicating how they were calculated

Our web collection on statistics for biologists contains articles on many of the points above.

Software and code

Policy information about availability of computer code

Data collection	Confocal images were taken by ZEN (black version 2.3), wide field fluorescence images were taken by Las X software (version: 3.8.2.27713).<br>Western Blot images were taken by ProteinSample FluorChem E system.
Data analysis	Interphase image analysis:<br>Foci counting analysis was performed by CellProfiler (4.2.1). Images were splited into single channel images, and segmentation was performed using IdentifyPrimaryObjects module with automatic thresholding and declamping. To analyze the foci structure, IF channel images were enhanced by calculating difference of two images applied with different Gaussian filter (σ=1, σ=2).<br>For colocalization analysis between IF and DNA FISH images, images were splited into single channel image, and IF, DNA FISH objects were called using IdentifyPrimaryObjects module in CellProfiler (4.2.1) independently. The colocalization between IF and DNA FISH objects were quantified through their presence at same location (x, y) as an object based colocalization analysis. Max projection was performed by FIJI-ImageJ (version 1.53t) before foci counting in Fig 3a.<br>Flow Cytometry data analysis:<br>The image in the schema in Extended Data Fig. 8f was generated by Beckman Coulter Kaluza software(version 2.1).<br>Cell viability assay data analysis was performed with GraphPad Prism (Version 9.1.0)<br>GRO-seq Data analysis:<br>The sequence data were mapped to human reference genome (hg38) using STAR (version 2.7.10b), HOMER (v4.11.1) was used for de novo transcript identification on each strand separately using the default GRO-seq setting. Reads with MAPQ values less than 10 were filtered using SAMtools (v1.8). Duplicate reads were removed using picard-tools. GRO-seq signal was converted to the bigwig format for visualization using deepTools bamCoverage (v3.3.1) with the following parameters: --binSize 10 --normalizeUsing CPM --effectiveGenomeSize 3209286105 --exactScaling.

KAS-seq Data analysis:
The sequence data were mapped to the hg38 assembly of the human genome using Bowtie with the following settings: -v 2-k 2-m 1--best--strata-X 1000. Duplicate reads were removed using picard-tools (version 1.99). MACS2 (v.2.1.1) was used for peak-calling with the following parameters: --broad -g hs --broad-cutoff 0.01 -q 0.01. Browser tracks are generated after normalizing to input using bamCompare default setting.
ChIP-seq Data analysis:
The sequence data were trimmed by Trimmomatic (v0.36) to remove adapter and then mapped to the hg38 assembly of the human genome using Bowtie2 with the following settings: --local --very-sensitive --phred33 -X 1000. Reads with MAPQ values less than 10 were filtered using SAMtools (v1.8). Duplicate reads were removed using picard-tools. CHIP-seq signal was convereted to the bigwig format for visualization using deepTools bamCoverage (v3.3.1) with the following parameters: --binSize 10 --normalizeUsing CPM --effectiveGenomeSize 3209286105 --exactScaling.

For manuscripts utilizing custom algorithms or software that are central to the research but not yet described in published literature, software must be made available to editors and reviewers. We strongly encourage code deposition in a community repository (e.g. GitHub). See the Nature Portfolio guidelines for submitting code & software for further information.

Data

Policy information about availability of data

All manuscripts must include a data availability statement. This statement should provide the following information, where applicable:

- Accession codes, unique identifiers, or web links for publicly available datasets
- A description of any restrictions on data availability
- For clinical datasets or third party data, please ensure that the statement adheres to our policy

GRO-seq, Ribo-Zero RNA-seq CHIP-seq, WGS and KAS-seq data generated in this study can be accessed by GEO under accession number GSE249657. AmpliconArchitect (AA) outputs and copy numbers of COLO320, GBM39 and PC3 can be accessed at https://ampliconrepository.org/project/6639560c48cbf4a5ccffad4d.

Research involving human participants, their data, or biological material

Policy information about studies with human participants or human data. See also policy information about sex, gender (identity/presentation), and sexual orientation and race, ethnicity and racism.

Reporting on sex and gender	not applicable
Reporting on race, ethnicity, or other socially relevant groupings	not applicable
Population characteristics	not applicable
Recruitment	not applicable
Ethics oversight	not applicable

Note that full information on the approval of the study protocol must also be provided in the manuscript.

Field-specific reporting

Please select the one below that is the best fit for your research. If you are not sure, read the appropriate sections before making your selection.

☒ Life sciences ☐ Behavioural & social sciences ☐ Ecological, evolutionary & environmental sciences

For a reference copy of the document with all sections, see nature.com/documents/nr-reporting-summary-flat.pdf

Life sciences study design

All studies must disclose on these points even when the disclosure is negative.

Sample size	No statistical methods were used to predetermine the sample size. For imaging, all the cells within 6-50 images were included into the analysis, except for cells on the edge, and experiments were repeated for 2 or more times independently. For western blot, experiments were performed at least 3 times with biologically independent replicates. For animal experiment, 8 mice was included in each group.
Data exclusions	No data we generated were excluded.
Replication	Experiments were repeated 2 or more times with similar outcome to ensure reproductivity. Animal experiment in Fig 4f was conducted once, with sample size of 8 within each group to ensure data reproductivity.
Randomization	To establish tumors, 1 million SNU-16 cells in 200 µL of a 1:1 mixture of PBS and Matrigel by subcutaneous injection into the right flank of 9-week-old female SCID beige mice. Animals (eight mice per group) were randomly assigned to treatment with vehicle, infigratinib (15 mg kg-1

PO QD), BBI-2779 (30 mg kg-1 PO Q2D), or the combination of BBI-2779 and infigratinib once average tumor volume was 286 (+/- 10) / mean (+/-SEM) mm3

Blinding	All data were collected using instruments without bias. Data within different groups were analyzed through same pipeline to ensure unbiased analysis. Blinding is not relevant to this study.

Reporting for specific materials, systems and methods

We require information from authors about some types of materials, experimental systems and methods used in many studies. Here, indicate whether each material, system or method listed is relevant to your study. If you are not sure if a list item applies to your research, read the appropriate section before selecting a response.

Materials & experimental systems

n/a	Involved in the study
☐	☒ Antibodies
☐	☒ Eukaryotic cell lines
☒	☐ Palaeontology and archaeology
☐	☒ Animals and other organisms
☒	☐ Clinical data
☒	☐ Dual use research of concern
☒	☐ Plants

Methods

n/a	Involved in the study
☐	☒ ChIP-seq
☐	☒ Flow cytometry
☒	☐ MRI-based neuroimaging

Antibodies

Antibodies used

ChIP-seq:
H3K36me3: Abcam, Cat# 9050, Lot # GR3459586-1

Western Blot:
pCHK1-S345 : Cell Signaling Technology, Cat# CST2348;
CHK1, Abcam, Cat# ab32531;
pRPA32/RPA2-Ser8 , Cell Signaling Technology, Cat# 54762S
γH2AX, Cell Signaling Technology, Cat# CST9718;
Vinculin , Cell Signaling Technology, Cat# CST13901
pFGFR2-Tyr653/654, Cell Signaling Technology, Cat# CST3476S
FGFR2 , Cell Signaling Technology, Cat# CST11835S;

Immunofluorescence:
γH2Ax, Millipore, Cat# 05-636,
pRPA2-S33, Novus Biological, Cat# NB100-544,
pCHK1S345, Invitrogen, Cat# PA5-34625,
53BP1, Novus Biological, Cat# NB100-304,
cyclin A, BD Bioscience, Cat# 611268,
pRNAPII S2/S4, Abcam, Cat# ab252855
Goat anti-rabbit IgG Alexa Fluor Plus 594; Thermo Fisher, #A32740s

Validation

All antibodies were validated by manufacture, and their validation statements are as follows:
H3K36me3: ChIP-grade antibody with over 900 references on abcam website and 760 reviews on Biocompare; validation based on overlap with gene body regions and depletion in non-coding regions of chromosomes; abcam validation by ChIP-qPCR

Western Blot:
pCHK1-S345, Cell Signaling Technology, Cat# CST2348; 841 citations.
CHK1, Abcam, Cat# ab32531; 11 citations. This antibody was validated by CHEK1 KO in A549 cells, using normal A549 as a control through Western Blot assay.
pRPA32/RPA2-Ser8 , Cell Signaling Technology, Cat# 54762S, 8 citations. Validated in HeLa and 293 cells, untreated or treated with UV (100 mJ/cm2, 2 hr recovery) through Western Blot.
γH2AX, Cell Signaling Technology, Cat# CST9718; 2071 citations. Validated in untreated or UV-treated 293 cells through Western Blot.
Vinculin , Cell Signaling Technology, Cat# CST13901, 426 citations.
pFGFR2-Tyr653/654, Cell Signaling Technology, Cat# CST3476S, 64 citations, validated in COS cells overexpressing human FGF receptor-1, untreated or calf intestine phosphatase (CIP)-treated, using Phospho-FGF Receptor (Tyr653/654) (55H2) Mouse mAb. Overexpression of human FGF receptor-1 results in constitutive activation of the receptors.
FGFR2 , Cell Signaling Technology, Cat# CST11835S; 36 citations. Validated by Western blot analysis of extracts from KATO III cells, transfected with 100 nM SignalSilence® Control siRNA (Unconjugated) #6568 (-) or SignalSilence®FGF Receptor 2 siRNA I #12600 (+).

Immunofluorescence:
γH2Ax, Millipore, Cat# 05-636, 3402 citations. 2 μg/ml of this antibody detected phosphorylated histone H2A.X in HeLa cells treated with 0.5 μM staurosporine for 4-6 hours.
pRPA2-S33, Novus Biological, Cat# NB100-544, 26 citations. 36 h post-IR treated OVCAR-8 cells with pro-resection genes (MRE1,

BRCA1 and WDR70) knockdown by specific siRNA, were subject to immunofluorescence detection of pRPA2-S33 detection in micronuclei and primary nuclei.
pCHK1S345, Invitrogen, Cat# PA5-34625, 5 citations. HeLa cells mock and treated with 100 J/m2 UVC and recover for 8 hrs were fixed in 4% paraformaldehyde at RT for 15 min and detected Phospho-CHK1-S345 by immunofluorescence.
53BP1, Novus Biological, Cat# NB100-304, 631 citations. Validated in 53BP1 knock out Hela cells by immunofluorescence.
cyclin A, BD Bioscience, Cat# 611268, 34 citations.
pRNAPII S2/S4, Abcam, Cat# ab252855, 5 citations. Validated in Hela cells treated with or without phosphatase at 37ºC for 2h by immunofluorescence.

Eukaryotic cell lines

Policy information about cell lines and Sex and Gender in Research

Cell line source(s)	GBM39ec, GBM39HSR and HK296 were patient derived neurosphere cell lines and were established as previously described. COLO320DM, COLO320HSR, SNU16, PC9, RPE1 were purchased from ATCC. The parental PC3 line was obtained from ATCC. PC3 DM and PC3 HSR lines were isolated by the Mischel lab through single cell expansions of the parental PC3 line.
Authentication	Cell lines obtained from ATCC were not authenticated. PC3-DM and PC3-HSR lines were isolated by the Mischel lab through single cell expansions of the parental PC3 line from ATCC and cell identity has been authenticated by STR profiling.
Mycoplasma contamination	Cells were tested negative for mycoplasma.
Commonly misidentified lines (See ICLAC register)	None of the cell line is listed in ICLAC register of Misidentified Cell lines.

Animals and other research organisms

Policy information about studies involving animals; ARRIVE guidelines recommended for reporting animal research, and Sex and Gender in Research

Laboratory animals	SCID beige mice (strain code 186; Envigo, Livermore, CA), 9 week-old
Wild animals	No wild animals were used in the study
Reporting on sex	This study does not imply any sex preference. Female SCID beige mice were utilized to minimize in fighting often found with non-liter mate male mice.
Field-collected samples	No field collected samples were used in the study
Ethics oversight	Animal experiments were performed in accordance with protocols approved by the CRADL Institutional Animal Care and Use Committee (Protocol #EB17-010-066)

Note that full information on the approval of the study protocol must also be provided in the manuscript.

Plants

Seed stocks	*Report on the source of all seed stocks or other plant material used. If applicable, state the seed stock centre and catalogue number. If plant specimens were collected from the field, describe the collection location, date and sampling procedures.*
Novel plant genotypes	*Describe the methods by which all novel plant genotypes were produced. This includes those generated by transgenic approaches, gene editing, chemical/radiation-based mutagenesis and hybridization. For transgenic lines, describe the transformation method, the number of independent lines analyzed and the generation upon which experiments were performed. For gene-edited lines, describe the editor used, the endogenous sequence targeted for editing, the targeting guide RNA sequence (if applicable) and how the editor was applied.*
Authentication	*Describe any authentication procedures for each seed stock used or novel genotype generated. Describe any experiments used to assess the effect of a mutation and, where applicable, how potential secondary effects (e.g. second site T-DNA insertions, mosiacism, off-target gene editing) were examined.*

ChIP-seq

Data deposition

☒ Confirm that both raw and final processed data have been deposited in a public database such as GEO.

☒ Confirm that you have deposited or provided access to graph files (e.g. BED files) for the called peaks.

Data access links *May remain private before publication.*	CHIP-seq data generated in this study can be accessed by GEO under accession number GSE249657

Files in database submission	COLO320, DM, input, rep1<br>COLO320, DM, input, rep2<br>COLO320, HSR, input, rep1<br>COLO320, HSR, input, rep2<br>COLO320, DM, H3K36me3, rep1<br>COLO320, DM, H3K36me3, rep2<br>COLO320, HSR, H3K36me3, rep1<br>COLO320, HSR, H3K36me3, rep2
Genome browser session (e.g. UCSC)	https://genome.ucsc.edu/s/gpw/hg38_ecDNA_TRC

Methodology

Replicates	Two biological replicates per sample
Sequencing depth	All ChIP-seq libraries were sequenced with paired end 75 bp reads<br><br>COLO320, DM, input, rep1, 23720427 total read pairs, 15028417 uniquely mapped reads, 99.79% overall alignment rate.<br>COLO320, DM, input, rep2, 16966012 total read pairs, 10405587 uniquely mapped reads, 99.83% overall alignment rate.<br>COLO320, HSR, input, rep1, 22223258 total read pairs, 13514979 uniquely mapped reads, 99.86% overall alignment rate.<br>COLO320, HSR, input, rep2, 17240809 total read pairs, 10740902 uniquely mapped reads, 99.81% overall alignment rate.<br>COLO320, DM, H3K36me3, rep1, 21404356 total read pairs, 13757242 uniquely mapped reads, 99.81% overall alignment rate.<br>COLO320, DM, H3K36me3, rep2, 27533870 total read pairs, 17330494 uniquely mapped reads, 99.83% overall alignment rate.<br>COLO320, HSR, H3K36me3, rep1, 19218975 total read pairs, 12114002 uniquely mapped reads, 99.81% overall alignment rate.<br>COLO320, HSR, H3K36me3, rep2, 16473728 total read pairs, 10468474 uniquely mapped reads, 99.80% overall alignment rate.
Antibodies	H3K36me3: Abcam, Cat# 9050, Lot # GR3459586-1
Peak calling parameters	no peak calling was needed
Data quality	no peak calling was needed
Software	Libraries were prepared using the NEBNext Ultra II DNA library prep kit (E7645) and sequenced by NovaSeq PE150. The sequence data were trimmed by Trimmomatic22 (v0.36) to remove adapter and then mapped to the hg38 assembly of the human genome using Bowtie219,20 with the following settings: --local --very-sensitive --phred33 -X 1000. Reads with MAPQ values less than 10 were filtered using SAMtools (v1.8). Duplicate reads were removed using picard-tools. CHIP-seq signal was convereted to the bigwig format for visualization using deepTools bamCoverage18 (v3.3.1) with the following parameters: --binSize 10 --normalizeUsing CPM --effectiveGenomeSize 3209286105 --exactScaling.

Flow Cytometry

Plots

Confirm that:

☒ The axis labels state the marker and fluorochrome used (e.g. CD4-FITC).

☒ The axis scales are clearly visible. Include numbers along axes only for bottom left plot of group (a 'group' is an analysis of identical markers).

☒ All plots are contour plots with outliers or pseudocolor plots.

☒ A numerical value for number of cells or percentage (with statistics) is provided.

Methodology

Sample preparation	Cell apoptosis was detected through flow cytometry using a FITC Annexin V Apoptosis Detection kit (BD bioscience, 556547). Cells were treated with inhibitor for the indicated time, and all the cells including floating cells were collected. After washing with PBS twice and cell number counting, cells were resuspended in 1X binding buffer, and stained with FITC Annexin V and PI for 15 min RT.
Instrument	BD LSRII follow cytometry (BD Biosciences)
Software	The image in the schema in Extended Data Fig. 8f was generated by Beckman Coulter Kaluza software
Cell population abundance	Cells were not sorted in this experiment.

Gating strategy

Major population cells were gated by FSC-A and SSC-A, and singlets were gated by FSC-A/FSC-H. Compensation degree was determined through single dye stained samples and non-stained control sample. All the singlets were further gated into Annexin V-FITC-/PI-; Annexin V+/PI-; Annexin V-/PI+; Annexin V+/PI+ populations based on non-stained control and single dye-stained samples.

☒ Tick this box to confirm that a figure exemplifying the gating strategy is provided in the Supplementary Information.
